# Laponite Lights Calcium Flickers by Reprogramming Lysosomes to Steer DC Migration for An Effective Antiviral CD8^+^ T‐Cell Response

**DOI:** 10.1002/advs.202303006

**Published:** 2023-08-28

**Authors:** Chenyan Li, Yangyang Hou, Minwei He, Liping Lv, Yulong Zhang, Sujing Sun, Yan Zhao, Xingzhao Liu, Ping Ma, Xiaohui Wang, Qianqian Zhou, Linsheng Zhan

**Affiliations:** ^1^ Institute of Health Service and Transfusion Medicine Beijing 100850 P. R. China; ^2^ BGI college, Henan Institute of Medical and Pharmaceutical Science Zhengzhou University Zhengzhou 450001 P. R. China

**Keywords:** calcium flickers, dendritic cell vaccine, laponite, lysosome reprogramming

## Abstract

Immunotherapy using dendritic cell (DC)‐based vaccination is an established approach for treating cancer and infectious diseases; however, its efficacy is limited. Therefore, targeting the restricted migratory capacity of the DCs may enhance their therapeutic efficacy. In this study, the effect of laponite (Lap) on DCs, which can be internalized into lysosomes and induce cytoskeletal reorganization via the lysosomal reprogramming–calcium flicker axis, is evaluated, and it is found that Lap dramatically improves the in vivo homing ability of these DCs to lymphoid tissues. In addition, Lap improves antigen cross‐presentation by DCs and increases DC‐T‐cell synapse formation, resulting in enhanced antigen‐specific CD8^+^ T‐cell activation. Furthermore, a Lap‐modified cocktail (Lap@cytokine cocktail [C‐C]) is constructed based on the gold standard, C‐C, as an adjuvant for DC vaccines. Lap@C‐C‐adjuvanted DCs initiated a robust cytotoxic T‐cell immune response against hepatitis B infection, resulting in > 99.6% clearance of viral DNA and successful hepatitis B surface antigen seroconversion. These findings highlight the potential value of Lap as a DC vaccine adjuvant that can regulate DC homing, and provide a basis for the development of effective DC vaccines.

## Graphical abstract

A novel, Laponite (Lap) optimized cytokine‐cocktail adjuvant for adoptive dendritic cell (DC) vaccine engineering is reported. Lap enhances DC homing and T cell infiltration through lysosomal reprogramming, promoting cytotoxic T cell immune responses against HBV infection. The calcium flicker‐cytoskeleton organization regulatory axis is proved to be the mechanism.

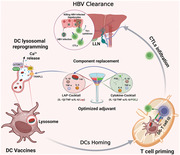

## 1 Introduction

Dendritic cells (DCs) are the most powerful specialized antigen‐presenting cells, bridging innate and adaptive immunity to activate naïve T cells.^[^ ^1^ ^]^ Successful ex vivo generation of DCs from their precursors has ushered in an era of adoptive DC vaccines.^[^ ^2^ ^]^ Currently, immunization with functionally competent DCs is an important treatment strategy for eradicating chronic infections^[^ ^1^ ^]^ and malignancies^[^ ^4^ ^]^ by overcoming the disease‐induced suppressive immune microenvironment and rebuilding the antigen‐specific T‐cell immune function of patients.^[^ ^5^ ^]^ To date, thousands of DC‐based clinical trials have been registered for the treatment of late‐stage cancers, autoimmune diseases, and chronic viral infections, including hepatitis B virus (HBV) and human immunodeficiency virus.^[^ ^6^ ^]^

A successful DC vaccine not only depends on efficient antigen upload and DC activation ex vivo, but also on efficient DC cell migration to lymph tissues in vivo to come into contact with T cells for antigen presentation.^[^ ^7^ ^]^ Adjuvants used during vaccine preparation activate DCs and reinforce their homing ability. Cytokine cocktail (C‐C), which is composed of interleukin‐1β (IL‐1β), IL‐6, tumor necrosis factor‐α (TNF‐α), and prostaglandin E_2_ (PGE_2_), is the most well‐established FDA‐approved DC vaccine adjuvant.^[^ ^8^ ^]^ Among these components, PGE_2_ is designed to promote DC homing and the other three cytokines are responsible for DC activation.^[^ ^9^ ^]^ With C‐C treatment, DCs can indeed be polarized toward a mature stage with upregulation of allostimulatory markers such as CD40 and CD80/86, and secretion of some T helper 1 (Th1) cytokines such as IL‐6 and TNF‐α.^[^ ^10^ ^]^ However, increasing evidence demonstrates that in response to C‐C stimulants, intricate intracellular signaling networks greatly reduce their respective effects.^[^ ^11^ ^]^ As a result, C‐C‐adjuvanted DCs lack essential cytokines for T‐cell activation such as IL‐12 and their homing ability (3–5%) is also greatly lower than expected,^[^ ^10^, ^12^ ^]^ resulting in only a 10–15% clinical response rate.^[^ ^13^ ^]^ These disappointing treatment results necessitate the optimization of C‐C components to overcome these drawbacks, where PGE_2_ is the most concerning. Although PGE_2_ can improve DC migration to some extent, its anti‐inflammatory nature dictates Th2 polarization^[^ ^14^ ^]^ and promotes the secretion of the immunosuppressive cytokine IL‐10,^[^ ^15^ ^]^ greatly hindering DC‐induced CD8^+^ T‐cell responses. Thus, identifying new adjuvant components that can promote DC migration and that do not interfere with responses to inflammatory stimuli is the key to improving the therapeutic effect of DC‐based immunotherapies.

The rapidly expanding field of nanovaccinology has greatly promoted the development of vaccine adjuvants.^[^ ^16^ ^]^ The unique physicochemical properties of nanoadjuvants, such as their large area‐to‐volume ratio, variable morphology, and rich surface functionalization modifications make them suitable for mimicking a naturally occurring pathogen invasion.^[^ ^17^ ^]^ Laponite (Lap) is a synthetic silicate clay with the empirical formula Na^+^_0.7_[(Si_8_Mg_5.5_Li_0.3_)O_20_(OH)_4_]^−0.7^. Its individual crystals are disc‐like structures that carry negative charges on both sides and weak positive charges on the edges, resulting in an overall negative charge.^[^ ^18^ ^]^ Lap has excellent adsorption and rheological properties and is typically used as an antistatic agent, protective coating, and thickener in the chemical industry. In recent years, Lap has been found to have potential for biomedical applications, including drug delivery, and tissue engineering, especially in vaccinology as antigen carriers.^[^ ^19^ ^]^ In addition, Lap induces oxidative stress and M1 polarization in macrophages, suggesting its potential immunoregulatory role.^[^ ^20^ ^]^ Recently, we accidentally found that well‐dispersed Lap nanosheets could be readily incorporated by DCs and most interestingly, induced sustained bright “calcium flickers” (Ca^2+^ transients) and intense cytoskeletal rearrangement in DCs. Moreover, evidence is mounting that both calcium transients and cytoskeletal reorganization are intimately connected with cell migration,^[^ ^21^ ^]^ where calcium transients, as a critical second messenger, provide migratory signals, and the cytoskeleton is the executor of cell movement.^[^ ^22^ ^]^ Hence, we hypothesized that Lap is a novel potential candidate as a DC‐targeted nanoadjuvant capable of improving DC homing ability and cytoskeletal rearrangement in a Ca^2+^‐dependent manner, thus increasing the therapeutic effect of DC vaccines by promoting DC‐T‐cell interaction.

To test this hypothesis, we systematically investigated the effects of Lap on DC maturation, migration, antigen presentation, and antiviral CD8^+^ T‐cell initiation in this study. The mechanisms of Lap‐enhanced DC homing were thoroughly investigated and a novel regulatory axis of lysosome reprogramming–calcium flicker–cytoskeleton reorganization is proposed in this study. Notably, we constructed a Lap‐modified C‐C by replacing PGE_2_ with Lap and demonstrated the superiority of the Lap@C‐C‐adjuvanted DC vaccine to induce CD8^+^ T cells. To the best of our knowledge, this is the first study to systematically explore the immunostimulatory effects of Lap and test its feasibility as an adjuvant for DC‐based immunotherapy. In addition, cost‐effective Lap raw materials and the simple preparation process, without any complex modification of Lap nanoadjuvants, will shed light on the construction of novel “easy‐to‐use” vaccine adjuvants.

## 2 Results and Discussion

**Figure 1:**
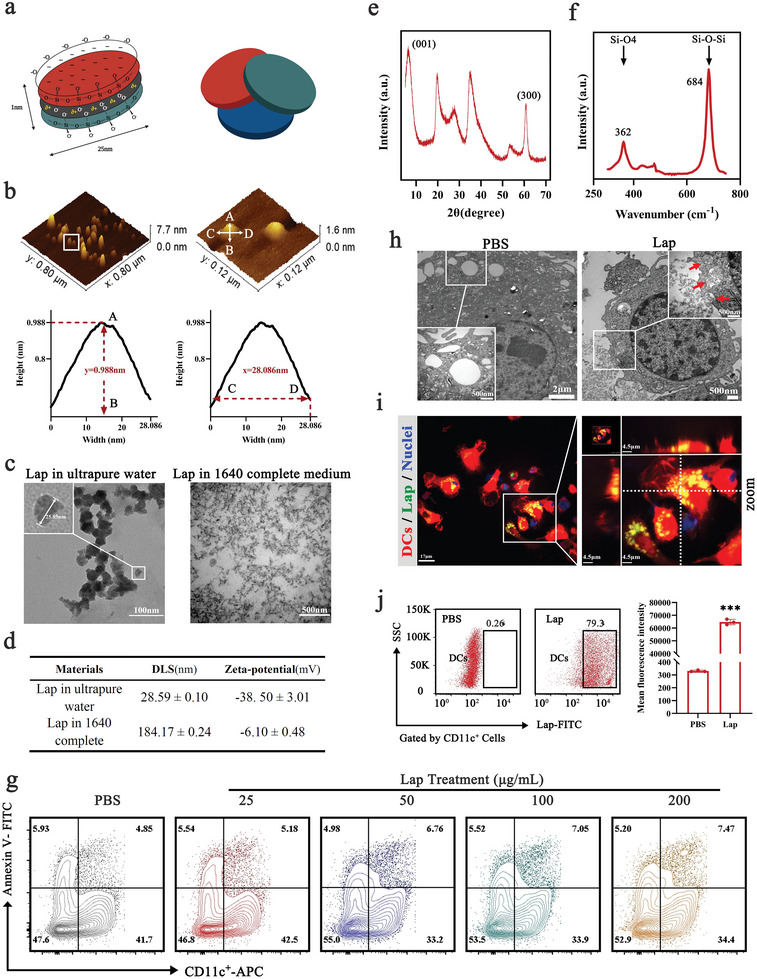
Characterization of Laponite (Lap) and its interaction with dendritic cells (DCs). a) Schematic of the layered structure of Lap. b) Atomic force microscopy (AFM) images of Lap. c) Transmission electron microscopy (TEM) images of Lap dispersion in water or RPMI‐1640 culture medium. d) Dynamic light scattering and zeta potential of Lap in different dispersions. e) X‐ray diffraction pattern of Lap. f) Fourier transform‐Raman spectra of Lap from 250 to 800 cm^−1^. g) Membrane integrity of DCs treated with Lap at a dose of 25–200 µg mL^−1^. h) TEM images of Lap‐treated DCs. The red arrows in the zoomed graph show the formation of phagocytic cups with intracellular transport of Lap. i) Confocal images of Lap locations in DCs. Red: DCs from tdTomato fluorescent protein transgenic mice; Green: fluorescein isothiocyanate (FITC)‐labeled Lap; blue: nuclei. j) Detection of Lap colocalized in DCs with flow cytometry (FCM). Data represent mean ± SD (n = 3). ***P < 0.001 compared with the phosphate‐buffered saline (PBS) group. Representative results from two or three replicates are shown.

The layered structure of Lap is shown in **Figure** **1a**. Each Lap consists of parallel sheets of Mg–O–Li sandwiched with Si–O–Si tetrahedral sheets in a 2:1 arrangement.^[^ ^23^ ^]^ The monolayer Lap was characterized by a diameter of ≈28 nm and a thickness of ≈1 nm using atomic force microscopy (AFM) (Figure 1b). Transmission electron microscopy (TEM) revealed that the morphology of Lap dispersed in ultrapure water was homogeneous with a disc‐like shape at a size consistent with the AFM data, whereas Lap in RPMI‐1640 complete medium self‐assembled into a 100–200 nm lamellar structure (Figure 1c). Figure 1d demonstrates that the hydrodynamic radii and zeta potential of Lap in RPMI‐1640 culture medium were either larger or higher than those in water, mainly due to the self‐aggregation of Lap in the saline solution and absorption of serum proteins. Its X‐ray diffraction pattern exhibited characteristic peaks at 2θ = 6.36° and 60.84°, which are related to the (001) and (300) crystallographic planes (Figure 1e). Raman spectra showed the basal profile of Lap with one band at 362 cm^−1^, assigned to the Si–O bending vibration, and another band at 684 cm^−1^, assigned to the Si–O–Si vibration (Figure 1f).^[^ ^24^ ^]^

Next, various doses of Lap were added to the medium and cocultured with DCs to test their cytotoxicity. Detection of the membrane integrity (Figure 1g) and the viability of Lap‐treated DCs (Figure S1, Supporting Information) demonstrated that DCs could tolerate Lap treatment well at a concentration ≤100 µg mL^−1^ with viability >95%. Furthermore, TEM observations (Figure 1h) confirmed that Lap was predominantly internalized by DCs and localized in phagosomes or lysosomes. Using Lap covalently modified by fluorescein isothiocyanate (FITC), confocal laser scanning microscopy (CLSM; Figure 1i) corroborated the TEM findings, confirming that Lap was located intracellularly and aggregated in the perinuclear regions of DCs. Moreover, flow cytometry (FCM) analysis detected a high percentage of DCs (>75%) with Lap colocalization (Figure 1j). These results indicated that Lap has a high affinity for DCs and is readily internalized by them.

**Figure 2:**
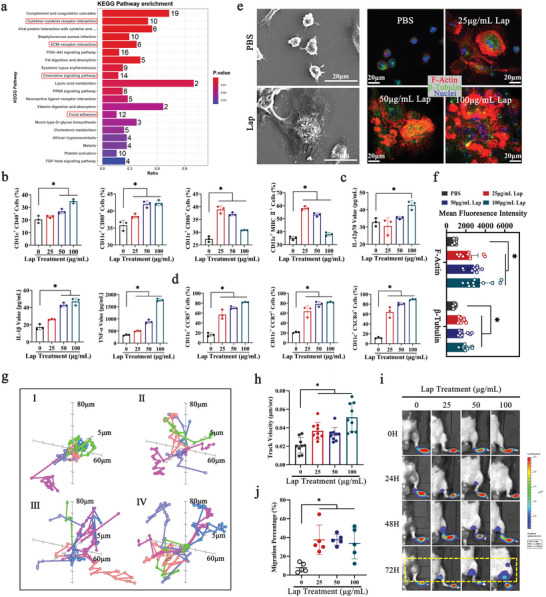
Lap enhanced DC maturation and migration ability. a) Kyoto Encyclopedia of Genes and Genomes (KEGG) pathway enrichment analysis of Lap‐upregulated proteins. The bar color indicates the *P* value and the top 20 KEGG pathways are shown. b) Detection of costimulatory molecules (CD40, CD80, CD86, and MHC II) expression on Lap‐treated DCs with FCM. Data represent mean ± standard deviation (SD; n = 3). c) The level of proinflammatory cytokine (interleukin [IL]−12p70, IL‐1β, and tumor necrosis factor‐α [TNF‐α]) secretion in Lap‐treated DCs analyzed using ELISA. Data represent mean ± SD (n = 3). d) FCM assays for chemokine receptor (CCR5, CCR7, and CXCR4) expression on Lap‐treated DCs. Data represent mean ± SD (n = 3). e) Morphology and cytoskeletal rearrangement of DCs after coincubation with Lap. Left panel: scanning electron microscopy (SEM) images of cell membrane structure and morphology changes in DCs induced by Lap; right panel: confocal imaging of cytoskeletal remodeling of DCs. Red: F‐actin; green: β‐tubulin. The mean fluorescence intensity of F‐actin and β‐tubulin was measured and displayed as f). Data represent mean ± SD (n = 10, “n” represents the number of fields observed per experimental group). g) The analysis of DC ex vivo movement with confocal laser scanning microscopy (CLSM). DCs were coincubated with I: PBS, II: 25 µg mL^−1^ Lap, III: 50 µg mL^−1^ Lap; and IV: 100 µg mL^−1^ Lap, respectively. The velocity of DCs was measured and displayed as h). Data represent mean ± SD (n = 9, “n” represents the number of cells observed per experimental group). i) Bioluminescence imaging of subcutaneously injected DCs homing to lymph nodes. The statistical data of the homing percentage are shown as mean ± SD (n = 5). *P < 0.05 compared with the PBS group. NS: *P* > 0.05 compared with the PBS group. Representative results from two or three replicates are shown.

To explore the biological effects of Lap on DCs, label‐free quantitative proteomics was employed to detect protein expression profiles before and after Lap treatment. More than 4500 proteins were analyzed, of which 590 were differentially expressed in Lap‐treated DCs (Figure S2, Supporting Information). Gene ontology enrichment analysis (Figure S3, Supporting Information) and Kyoto Encyclopedia of Genes and Genomes (KEGG) pathway enrichment analysis (**Figure** **2a**) demonstrated that the upregulated differentially expressed proteins (DEPs) were mainly associated with chemokine‐mediated signaling pathways, inflammatory responses, and cell migration, suggesting that Lap exerted an enormous effect on DC maturation and migration. We then examined the expression levels of costimulatory molecules on DCs, which is a hallmark of DC maturation and a prerequisite for initiating T‐cell immune responses.^[^ ^25^ ^]^ As expected, the expression levels of CD40, CD80, CD86, and major histocompatibility complex class II molecules (MHC II) on the surface of Lap‐treated DCs (Lap‐DCs) were considerably upregulated compared with those on untreated DCs (PBS‐DCs) (Figure 2b). In addition, IL‐12p70, IL‐1β, and TNF‐α levels increased in a dose‐dependent manner with Lap treatment, reaching their peak at 100 µg mL^−1^ of Lap (Figure 2c). These data confirm that Lap treatment induces significant inflammatory responses in DCs.

DC migration is tightly regulated by a large variety of signals, among which the receptor–chemokine axes and cytoskeleton play important roles.^[^ ^26^ ^]^ The chemokine receptors CCR5, CCR7, and CXCR4 on DCs, which are important for tissue‐resident DCs to overcome extracellular matrix barriers and enter lymph vessels, were detected by fluorescence‐activated cell sorting (Figure 2d).^[^ ^27^ ^]^ CCR5, CCR7, and CXCR4 were strikingly upregulated after Lap treatment, with respective increases of 4.6, 3.6, and 6.9‐fold compared with PBS‐DCs, suggesting that Lap can enhance DC chemotaxis for multiple chemokines, including MIP/RANTES (CCR5 ligand), CCL19/21 (CCR7 ligand), and CXCL12 (CXCR4 ligand). In addition to mobilization signals, DCs must undergo robust shape changes and cytoskeletal reorganization for migration.^[^ ^28^ ^]^ Scanning electron microscopy (SEM) images showed that Lap treatment caused significant morphological changes in DCs, resulting in a shuttle shape with a corrugated membrane surface and a substantial increase in membrane protrusion (Figure 2e, left panel). In addition, Lap increased the fluorescence intensity of F‐actin and β‐tubulin staining in DCs, improved the formation of focal adhesions, and promoted the aggregation of microfilaments in a dose‐dependent manner (Figure 2e, right panel, and Figure 2f), suggesting an enhanced cytoskeletal rearrangement of Lap‐DCs. The cytoskeleton is the executor of migratory actions.^[^ ^29^ ^]^ To further investigate the cytoskeleton‐associated functional changes in Lap‐treated DCs, live‐cell time‐lapse imaging was employed to directly track the in vitro motility of DCs at 3 min‐intervals over an 8 h duration (Figure 2g). Critical parameters tightly linked to DC mobility were calculated. The total movement distance (Figure S4, Supporting Information) and velocity (Figure 2h) of Lap‐treated DCs, especially at the highest dose of Lap, were significantly longer and faster than those of their untreated counterparts by up to 1.8‐fold and 1.6‐fold, respectively. These data demonstrate that Lap treatment enhances DC chemotaxis and migration, indicating an improved in vivo homing ability of DCs to lymphoid tissue. To analyze Lap‐enhanced DC homing directly, bioluminescence imaging (BLI) combined with a footpad injection mouse model was employed to monitor the homing of firefly luciferase (Fluc)‐expressing DCs from the subcutaneous footpad to the adjacent popliteal lymph node (PLN) and inguinal lymph node (ILN). The light intensity (SI) emitted by Fluc reflects the number of DCs. The results showed that untreated DCs were mainly retained in the footpad at all detected time points. In contrast, Lap‐treated DCs had already migrated to the PLNs as early as 24 h, accumulating at high percentages at 72 h post‐injection (Figure 2i). The homing percentage of the DCs was quantified using the following formula: DC homing rate = SI (ILN + PLN)/SI (ILN + PLN + footpad). Only 7.9 ± 4.9% of the untreated DCs migrated to the PLNs and ILNs (Figure 2j). However, the homing percentage of DCs in the Lap‐treated groups at 25, 50, and 100 µg mL^−1^ Lap reached 37.5 ± 15.8%, 37.6 ± 5.7%, and 33.7 ± 16.6%, respectively, which demonstrates that Lap treatment increased the proportion of homed DCs by at least 4.3 fold. Furthermore, GFP expressing‐DCs were injected to the footpad of mice and PLNs were isolated at 72 h to confirm the homing of Lap‐treated DCs (Figure S5, Supporting Information). The result corroborated with the BLI detection (Figure 2i), confirming Lap indeed promoted DC migration.

**Figure 3:**
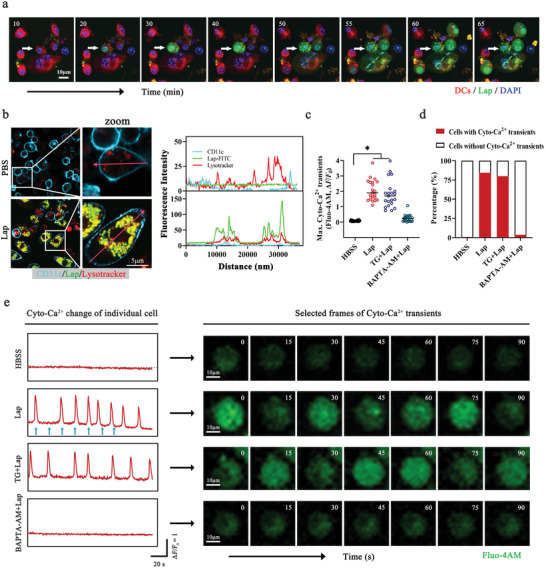
Lap‐induced calcium flickers in DCs. a) Representative dynamic imaging of live DCs treated with Lap. Red: DCs stained with CellTracker Deep Red Dye; green: FITC‐labeled Lap. The numbers on the graphs indicate the time in minutes. See also Video S1 (Supporting Information). b) Colocalization analysis of Lap location on DCs. Left panel: CLSM images of DCs treated with Lap for 1 h. Turquoise: DC surface marker CD11c; green: FITC‐labeled Lap; red: LysoTracker. Right panel: fluorescence intensity statistics along the cherry arrows. c) DCs Cyto‐Ca^2+^ transient amplitudes were quantified by measuring the change in maximum fluorescence intensity of Fluo‐4AM; statistics are shown as mean ± SD (n = 21, “n” represents the number of cells observed per experimental group). d) Percentage of DCs exhibiting Cyto‐Ca^2+^ transients in the Hanks’ Balanced Salt Solution (HBSS) group as well as the Lap‐treated group after inhibitor pretreatment. HBSS group: n = 119; Lap group: n = 153; TG + Lap group: n = 130; BAPTA‐AM + Lap group: n = 141, “n” represents the number of cells observed per experimental group. e) Dynamic monitoring of Cyto‐Ca^2+^ in DCs. Cells were challenged with Lap ± Thapsigargin (TG, microsomal Ca^2+^‐ATPase inhibitor, 1 µM) and BAPTA‐AM (Cyto‐Ca^2+^ chelator, 10 µM). Left panel: Representative tracking of the Cyto‐Ca^2+^ dynamics of DCs labeled with Fluo‐4AM. Blue arrows: Time points of the displayed Cyto‐Ca^2+^ transient frames. Right panel: selected frames of Cyto‐Ca^2+^ transients in DCs. The numbers on the graphs indicate the time in seconds. See also Video [Link], [Link], [Link], [Link] (Supporting Information). *P < 0.05 compared with PBS or HBSS group. Representative results from two or three replicates are shown.

From the above data, the most dramatic functional changes in DCs induced by Lap were increased migration and homing abilities. Recently, the molecular mechanisms controlling DC migration have been described in detail. Multiple intracellular signaling pathways comprise a complex regulatory network that ultimately outputs the order of cell movement by controlling cytoskeletal reorganization.^[^ ^7^, ^30^ ^]^ To elucidate the signaling transduction pathways in Lap‐enhanced DC migration, we analyzed the proteomic data in depth and paid special attention to the pathways dominating cellular motility. Numerous proteins involved in vesicular transport and Ca^2+^ homeostasis were significantly upregulated in Lap‐treated DCs (Figure S6, Supporting Information). Homeostasis of Ca^2+^, which is an important secondary messenger, is essential for DC migration as it controls the phosphorylation of a variety of signaling proteins such as Rac1, Cdc42, and PKC upstream of cytoskeletal components, including F‐actin and focal adhesion.^[^ ^31^ ^]^ To play this role, intracellular Ca^2+^ must be tightly regulated; Ca^2+^ pulses and spikes should occur at the right place and time, selectively activating numerous downstream signaling targets.^[^ ^32^ ^]^ Before testing whether Lap acts on Ca^2+^ homeostasis, we characterized the process of Lap entering DCs to capture Lap‐induced Ca^2+^ transients. Live‐cell time‐lapse imaging confirmed that the internalization event occurred ≈10 min after Lap treatment (**Figure** **3a**, Video S1, Supporting Information) and was mainly localized in the lysosomes (Figure 3b). Therefore, the 10th min after the Lap addition was selected as the time point for Ca^2+^ detection. To visualize Ca^2+^ transients, DCs uploaded with the cytoplasm‐Ca^2+^ indicator Fluo‐4AM were sequentially imaged every 320 ms. As expected, bright fluorescence signals (intensity: 8.76 ± 4.35) (Figure 3c) were detected with a high proportion of Lap‐treated DCs (84.3%) (Figure 3d), which presented classic pulse‐like flashing with an interval time of ≈30 s (Figure 3e, Videos [Link], [Link], [Link], [Link], Supporting Information). No significant light signal was observed from untreated DCs or DCs treated with both Lap and the Ca^2+^ chelator BAPTA‐AM, confirming the specificity of Lap‐induced Ca^2+^ transients in DCs. In addition, Lap could still induce robust Ca^2+^ transients even when DCs were pretreated with thapsigargin, which specifically inhibits endoplasmic reticulum Ca^2+^ mobilization.^[^ ^33^ ^]^ This suggests that Lap‐modified Ca^2+^ homeostasis was not dominated by the Ca^2+^ pool in the endoplasmic reticulum. Furthermore, the intracellular F‐actin rearrangement in Lap‐treated DCs with pre‐blocked Ca^2+^ transients was visualized (Figure S7, Supporting Information), showing that the Ca^2+^ chelator remarkably inhibited Lap‐induced F‐actin reorganization, confirming that Ca^2+^ homeostasis is indeed involved in Lap‐enhanced DC migration.

**Figure 4:**
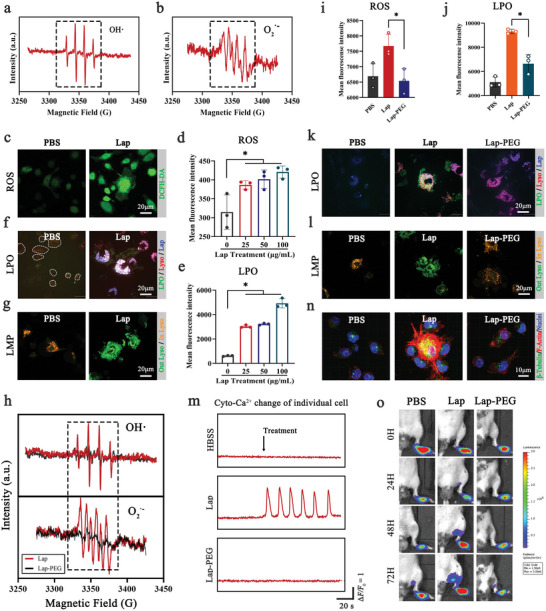
Lap promoted DC migration via free radical‐induced lysosomal reprogramming. a, b) Electron paramagnetic resonance (EPR) spectra of hydroxyl radicals and superoxide radicals on Lap. c) Reactive oxygen species (ROS) accumulation in DCs after Lap coincubation was detected using the 2′−7′ dichlorofluorescein diacetate (DCFH‐DA) probe. d) FCM analysis of ROS level in DCs after Lap co‐incubation. Data showed as mean ± SD (n = 3, “n” represents three independent replicate samples per experimental group). e) FCM analysis of LPO level in DCs after Lap co‐incubation. Data showed as mean ± SD (n = 3, “n” represents three independent replicate samples per experimental group). f) LPO accumulation in DCs after Lap co‐incubation was detected using the Liperfluo probe. Green: LPO indicator; Red: LysoTracker; Blue: rhodamine‐labeled Lap. g) Detection of DC lysosomal membrane permeability (LMP) after Lap coincubation using acridine orange (AO) staining. Orange: AO is located in the lysosomes. Green: AO escapes from the lysosomal lumen to the cytosol. h) EPR spectra of free radicals on Lap and Lap‐polyethylene glycol (PEG). i) The effect of PEGylation on Lap‐induced ROS elevation was analyzed using FCM. Data represent mean ± SD (n = 3). j) The effect of PEGylation on Lap‐induced LPO accumulation was analyzed by FCM. Data represent mean ± SD (n = 3, “n” represents three independent replicate samples per experimental group). k) CLSM images of the effect of PEGylation on Lap‐induced LPO accumulation. l) CLSM images of PEGylation interference with Lap‐stimulated LMP. m) Dynamic monitoring of Cyto‐Ca^2+^ transients of Lap‐DCs and Lap‐PEG‐DCs. The percentages of DCs with Ca^2+^ changes are shown below. n) Cytoskeletal rearrangement of DCs after coincubation with Lap and Lap‐PEG. Red: F‐actin; green: β‐tubulin; blue: nuclei. The mean fluorescence intensity of F‐actin and β‐tubulin was measured and displayed. o) Bioluminescence imaging of DCs homing to lymph nodes after coincubation with Lap and Lap‐PEG (n = 5). *P < 0.05 between the two groups indicated. Representative results from two or three replicates are shown.

Next, we investigated the effect of Lap on intracellular Ca^2+^. When detecting Ca^2+^ transients, we used a Ca^2+^‐free medium for DC culture; thus, the increased cytosolic Ca^2+^ level did not originate from extracellular Ca^2+^ influx, and Lap mobilized intracellular Ca^2+^ stores.^[^ ^22^ ^]^ In addition, the endoplasmic reticulum Ca^2+^ pool is not the main source of Ca^2+^ as it is insensitive to pretreatment with the thapsigargin (Figure 3e), indicating an alternative intracellular compartment is responsible for Lap‐induced Ca^2+^ signals. Lysosomes, as “housekeeping” organelles, are responsible for degradation and recycling in the cell.^[^ ^34^ ^]^ But studies have emphasized the ability of lysosomes to store Ca^2+^ with intraluminal Ca^2+^ of approximately 500 µM and to participate in calcium signaling processes.^[^ ^30^, ^33^, ^35^ ^]^ In particular, Ca^2+^ released from lysosomes can specifically induce cytoskeletal rearrangement and promote rapid and directional migration of cells.^[^ ^22^, ^28^ ^]^ These findings encouraged us to investigate whether lysosomes participate in Lap‐induced Ca^2+^ regulation in DCs, thereby enhancing DC migration. First, we found that Lap entered DCs mainly through clathrin‐mediated endocytosis, as treatment with chlorpromazine hydrochloride substantially inhibited their internalization (Figure S8, Supporting Information). Therefore, early endosomes and later lysosomes are the main endomembrane systems that are in direct and long‐term contact with the internalized Lap. Hence, direct Lap exposure makes lysosomes highly susceptible to the physicochemical properties of Lap. Using electron paramagnetic resonance (EPR) spectroscopy, we detected abundant Lap‐generated hydroxyl and superoxide radicals in the aqueous phase (**Figure** **4a,b**), indicating that Lap is highly likely to cause oxidative stress in DCs. Consistently, the reactive oxygen species (ROS) level in Lap‐treated DCs increased by ≈1.3‐fold compared with that in untreated DCs (Figure 4c,d). In particular, lipid peroxidation (LPO) in Lap‐treated DCs increased in a dose‐dependent manner and was up to 7.9‐fold higher than the control group (Figure 4e). Moreover, the highly selective colocalization of LPO reporter probes and LysoTracker fluorescent dyes revealed that lysosomes were the main source of Lap‐induced LPO accumulation (Figure 4f, Figure S9, Supporting information). Given that LPO can greatly affect the fluidity and permeability of the membrane structure, the lysosomal membrane permeability (LMP) was further analyzed using acridine orange (AO) staining (Figure 4g) and galectin‐3 (LGALS3) puncta assay (Figure S10, Supporting information). The shift from orange to green fluorescence signals after AO staining and the formation of bright galectin‐3 puncta indicated that Lap indeed increased LMP. In addition, the significantly enlarged lysosomes as shown in Figures 3b and 4f also correspond to the typical features occurred during LMP. In combination, those data highlight the internalized Lap was specifically located in lysosomes and then caused intense lysosomal stress due to the generated radicals, finally enhancing lysosomal membrane permeability and involving in the regulation of DCs migration.

As indicated above, lysosomes are assumed to be the stores from which Lap mobilizes Ca^2+^ release. To prove this assumption and test whether Ca^2+^ mobilization is related to Lap‐induced lysosomal stress, we constructed polyethylene glycol (PEG)‐modified Lap (Lap‐PEG), which cannot generate radicals. EPR spectroscopy data showed that both the hydroxyl and superoxide radicals generated by Lap dramatically decreased with PEGylation (Figure 4h). Simultaneously, PEG modification remarkably mitigated the Lap‐induced ROS levels (Figure 4i), lysosomal LPO accumulation (Figure 4j,k), and the extent of LMP (Figure 4l). Notably, the alleviation of lysosomal stress can be largely attributed to the loss of radicals in Lap, as inductively coupled plasma mass spectrometry analysis excluded the interference of PEGylation on Lap internalization (Figure S11, Supporting Information). Based on these results, we explored the relationship between lysosomal stress and Ca^2+^ release. The loss of LPO accumulation and LMP occurrence in Lap‐PEG‐treated DCs dramatically downregulated Lap‐induced Ca^2+^ transients, reaching the baseline level, comparable with untreated DCs (Figure 4m; Figure S12, Supporting Information). In line with this, the levels of transcription factor EB (TFEB) and Rab7b, both critical signaling proteins downstream of the lysosomal Ca^2+^ pathway,^[^ ^28^ ^]^ simultaneously decreased in the Lap‐PEG group (Figure S13, Supporting Information). Similar tendencies were observed for DC cytoskeletal rearrangement (Figure 4n) and in vivo homing (Figure 4o; Figure S14, Supporting Information). Moreover, the direct link between ROS and Ca^2+^ mobilization following Lap treatment is also confirmed with N‐acetyl‐L‐cysteine (NAC, a direct ROS inhibitor) treatment (Figure S15, Supporting Information), in which treating with NAC significantly reduces the Ca^2+^ flickering induced by Lap. In summary, once Lap is incapacitated to generate radicals, it loses the ability to induce lysosomal stress, accompanied by the loss of Ca^2+^ mobilization, and finally decrease of Lap‐induced DC homing, suggesting a novel regulatory axis of Lap‐induced lysosomal reprogramming, Ca^2+^ mobilization, and DC migration.^[^ ^31^, ^32^ ^]^ Recently, several studies have designed nanovaccines targeting Ca^2+^‐related specific immune activation. However, most of these are calcium‐based nanomaterials (e.g., CaP, CaO_2_, CaCO_3_, and Ca_10_(PO_4_)_6_(OH)_2_) with the objective of introducing exogenous Ca^2+^.^[^ ^36^ ^]^ One critical limitation of this strategy is the risk of Ca^2+^ overload, which can kill target cells.^[^ ^37^ ^]^ The most interesting finding of our study is that endogenous Ca^2+^ mobilization can be achieved by Ca^2+^‐free nanoparticles, where oxidative stress‐induced lysosomal reprogramming plays a central role. Limited by the lack of tools for specifically investigating lysosomal Ca^2+^ signaling, such as lysosomal channel agonists or a direct inhibitor of lysosomal Ca^2+^ uptake, the precise mechanism(s) of how Lap releases Ca^2+^ and how intracellular Ca^2+^ filling occurs remains uncertain. However, lysosomal Ca^2+^ efflux may likely be due, at least in part, to increased LMP. First, Lap‐induced LMP allowed the passive efflux of AO fluorescent probes (Figure 4g); thus, smaller Ca^2+^ ions can also readily pass. Second, Lap induced the Ca^2+^ transients with a lag time of ≈10 mins and most importantly, its response kinetics resemble “regenerative” or “pulse” responses (a comparatively sharp up‐stroke after a “pacemaker” rise). These characteristics bear a striking similarity to the Ca^2+^ release induced by GPN (glycyl‐L‐phenylalanine 2‐naphthylamide). As a lysosomotropic agent, GPN has been shown to release Ca^2+^ by inducing LMP.^[^ ^38^ ^]^ Hence, LMP is thought to play a role in Lap‐induced Ca^2+^ mobilization. However, another point of uncertainty is whether LMP is the only factor involved. Lysosomes are H^+^‐rich organelles, and their low pH allows Lap degradation into various ionic components, including silicic acid, magnesium ions, and lithium ions (Table S1, Supporting Information), which can in turn induce changes in H^+^ content. In addition, lysosomes possess many Ca^2+^‐permeable channels and exchangers such as the Ca^2+^‐H^+^ exchanger.^[^ ^39^ ^]^ Therefore, it is possible that Lap degradation plays a supporting role in Ca^2+^ efflux or filling. In summary, although the precise mechanism of how Lap mobilizes Ca^2+^ requires additional experimental confirmation, it is certain that Lap treatment can induce dramatic endogenous Ca^2+^ influx in DCs, which efficiently enables DCs to drain into LNs to contact T cells.

**Figure 5:**
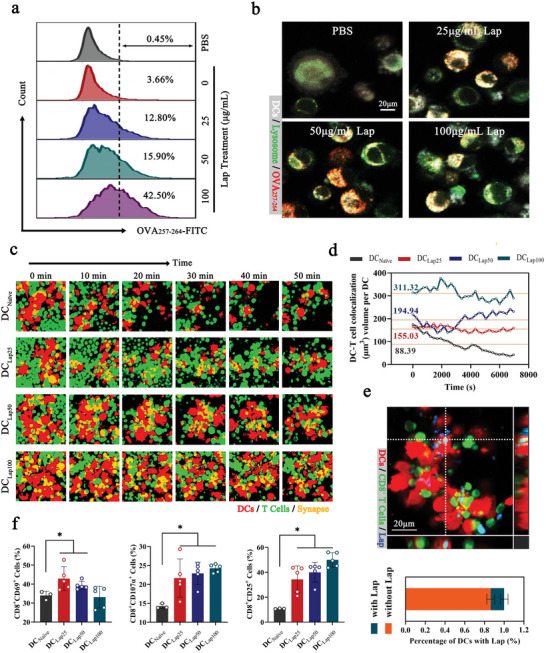
Lap enhanced the in vitro CD8^+^ T‐cell priming abilities of DCs. a) FCM analysis of Lap‐DCs coincubated with FITC‐labeled OVAp for 12 h. The representative CLSM images are shown in b). c) Time‐lapse imaging of dynamic interactions between DCs and T cells. Red: DCs expressing tdTomato fluorescent protein; green: CellTracker Deep Red Dye‐labeled CD8^+^ T cells from OT‐I T‐cell receptor transgenic mice; yellow: DC‐T cell synapses. d) The DC‐T‐cell colocalization volume of DC‐T cells over 120 min. e) Proportion of Lap‐containing DC‐T‐cell synapses. Upper panel: CLSM image of colocalization of Lap, DCs, and T cells. Red: DCs expressing tdTomato fluorescent protein; green: CellTracker Deep Red Dye‐labeled CD8^+^ T cells from OT‐I T‐cell receptor transgenic mice; blue: FITC‐labeled Lap. Bottom panel: Statistical analysis of the percentage of DCs with Lap. Data represent mean ± SD (n = 8, “n” represents the number of fields observed per experimental group). f) FCM assays for activation of CD8^+^ T cells coincubated with Lap‐DCs, including CD25, CD69, and CD107a. Data represent mean ± SD (n = 5). *P < 0.05 compared with DC_Naïve_ group. Representative results from two or three replicates are shown.

The ultimate goal of DCs is to initiate T‐cells. Mounting evidence suggests that exogenous antigen transfer to DCs and their surrogate cross‐presentation on MHC I are paramount in orchestrating antigen‐specific CD8^+^ T‐cell responses.^[^ ^40^ ^]^ To determine whether Lap affects DC antigen cross‐presentation, DCs were pulsed with ovalbumin 257–264 (OVA_257–264_, SIINFEKL), an octapeptide specifically presented by MHC I, and the H‐2Kb‐OVA_257–264_ complexes on the DCs were quantified. As shown in **Figure** **5**a, Lap treatment increased OVA_257–264_ presentation in a dose‐dependent manner, with an ultimate increase of 3.2‐fold at a dose of 100 µg mL^−1^ of Lap (Figure S16, Supporting Information).

A major mechanism of antigen cross‐presentation is the escape of exogenous antigens from endosomes or lysosomes to the cytosolic proteasome before peptide loading into the endoplasmic reticulum.^[^ ^41^ ^]^ Utilizing FITC‐covalently modified OVA_257–264_ for antigen challenge, it was found that most of the antigens were enriched in the lysosomes of untreated DCs, with less leakage into the cytoplasm (Figure 5b). In sharp contrast, Lap treatment, especially at the highest dose, dramatically increased the cytoplasmic distribution of OVA_257–264_, indicating that Lap increased antigen presentation, likely by facilitating exogenic antigen escape from lysosomes. Combined with the occurrence of LMP (Figure 4 g), it was deduced that Lap‐induced antigen leakage was highly related to enhanced lysosomal membrane permeability.

In addition to antigen recognition, T‐cell activation requires the formation of a stable DC‐T‐cell membrane junction‐immune synapse (IS),^[^ ^5^ ^]^ which can be visualized by the containing of DCs and T‐cells. CD8^+^ T‐cells from OT‐I TCR transgenic mice were cocultured with OVA_257–264_‐pulsed DCs, and their interactions were recorded by time‐lapse imaging. From the 90 min recording, distinctly larger and more sustainable DC‐T‐cell clusters were observed in the Lap‐treated groups than in the untreated groups (Figure 5c; Figure S17, Supporting Information). Statistical analysis revealed that Lap treatment allowed the DC‐T‐cell colocalization volume to increase by approximately 3.5‐fold (100 µg mL^−1^ group) (Figure 5d). Furthermore, Lap mainly colocalized with DCs, with less binding to T‐cells (Figure 5e, upper panel), excluding the possibility that Lap physically bridges the DC and T‐cell membranes to a large extent. Consistently, <15% of DC‐T‐cell colocalized areas contained Lap (Figure 5e, lower panel), confirming that the existence of Lap would not interrupt molecule pairing between DCs and T‐cells. In addition, Lap promoted DC‐T‐cell IS formation mainly by inducing Ca^2+^ influx (Figure S18, Supporting information), and Ca^2+^ enhanced cytoskeletal organization likely contribute to this process with regard to the critical role that cytoskeletons play in supporting the formation of the DC‐T‐cell synapse interface.

Upon activation, T‐cells induce and sequentially express multiple activation markers, including early CD69, CD25, and CD107a (LAMP‐1), which reflects the cytotoxic activity of T‐cells.^[^ ^42^ ^]^ As shown in Figure 5f, Lap‐treated DCs exhibited an improved capacity to activate T‐cells, resulting in significantly elevated CD69, CD25, and CD107a levels in CD8^+^ T‐cells. In addition, the dose of Lap used for treatment was correlated with the manner in which DC activated T‐cells, that is, acute or chronic activation. With the increase in exposure to Lap, DCs gained an increasingly strong capacity to activate T‐cells characterized by decreasing CD69 (early activation marker) and increasing CD25 (late activation marker) levels in activated CD8^+^ T‐cells, indicating that DCs treated with high‐dose Lap enabled T‐cells to enter an acute activation stage with increased expression of late activation markers. In summary, the above findings consistently demonstrated that Lap plays an immunostimulatory role in DC‐T‐cell interactions, mainly by promoting DC antigen cross‐presentation and increasing DC‐T‐cell IS formation, which together results in enhanced antigen‐specific CD8^+^ T‐cell activation.

**Figure 6:**
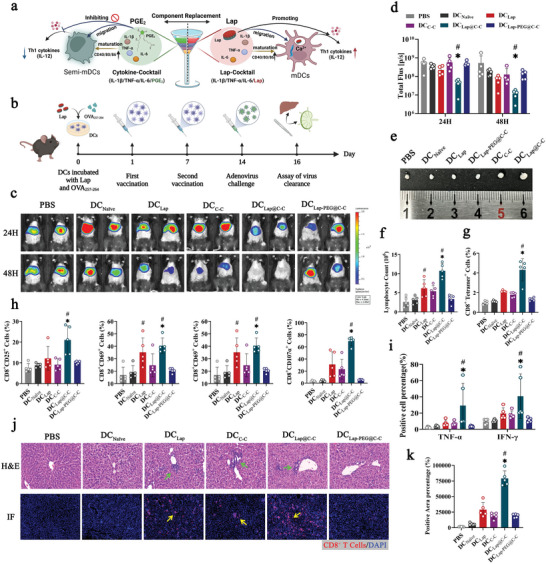
In vivo CD8^+^ T cells priming and antiviral effect of Lap@cytokine cocktail (C‐C)‐adjuvanted DC vaccine. a) Schematic of the composition of Lap‐modified C‐C. b) Schematic diagram of the immunization schedule for the adenoviral serotype‐5 vector containing OVA_257‐264_ and firefly luciferase (Ad‐OVAp‐Fluc) challenge model. c, d) Representative images of bioluminescence imaging and statistical analysis of the light intensity of Ad‐OVAp‐Fluc infection in the liver (PBS: Mice treated with PBS; DC_Naïve_: Mice vaccinated with Naïve imDCs; DC_Lap_: Mice vaccinated with Lap‐adjuvanted DCs; DC_C‐C_: Mice vaccinated with C‐C‐adjuvanted DCs; DC_Lap@C‐C_: Mice vaccinated with Lap@C‐C‐adjuvanted DCs; DC_PEG@C‐C_: Mice vaccinated with PEG@C‐C‐adjuvanted DCs). e) After monitoring, mice were euthanized to collect liver lymph nodes (LLNs), and their sizes were recorded. f, g) The proportions of total and OVA_257‐264_‐specific CD8^+^ T cells in LLNs were analyzed. h) The expression of CD8^+^ T activation markers was detected by FCM, including CD25, CD44, CD69, and CD107α. i) The release levels of proinflammatory cytokines TNF‐α and interferon‐γ. j) Representative images of pathology analysis of the liver. Upper panel: hematoxylin & eosin (H&E) staining of liver sections. Bottom panel: Immunofluorescence staining of liver sections. Green and yellow arrows indicate the infiltration of inflammatory cells and CD8^+^ T cells, the latter is quantified in k). Data represent mean ± SD (n = 5, “n” represents the number of fields observed per experimental group). g) *P < 0.05 compared with the DC_Naïve_ group. #*P* < 0.05 compared with the DC_C‐C_ group. Representative results from two or three replicates are shown.

Given that Lap enables DCs to come into contact with T‐cells and enhances DC‐T‐cell interplay, it may be feasible as an adjuvant in DC immunotherapy. Here, a novel C‐C optimization scheme is proposed by replacing PGE_2_ with Lap to form Lap‐modified C‐C (Lap@C‐C) (**Figure** **6a**). Lap@C‐C was designed to balance the maturation and migration of DCs by breaking the potential immunosuppressive barrier against PGE_2_.^[^ ^43^ ^]^ Elevated secretion of IL‐12p70, a critical cytokine for initiating T‐cell responses, from Lap@C‐C‐adjuvated DCs compared to that from “C‐C”‐adjuvated DCs was observed (Figure S19, Supporting Information). Next, a serotype‐5 recombinant adenovirus containing OVA_257–264_ and firefly luciferase (Fluc^+^; Ad‐OVA_257–264_‐Fluc) was constructed. Intravenous administration of Ad‐OVA_257–264_‐Fluc resulted in liver infection, which allowed quantification of the OVA‐specific CD8^+^ T‐cell response by imaging viral clearance.^[^ ^44^ ^]^ OVA_257–264_‐pulsed DCs were stimulated with various adjuvants and injected into the footpads of mice for subcutaneous vaccination on days 1 and 7 (Figure 6b). On day 14, the mice were challenged with adenovirus serotype 5 vector carrying OVAp and Fluc^+^ (Ad‐OVAp‐Fluc) and monitored using BLI to detect viral clearance. Sequential imaging (Figure 6c) and its statistical data (Figure 6d) revealed all DC vaccines adjuvanted by respective “C‐C,” “Lap” (monocomponent), or “Lap@C‐C” resulted in dramatical bioluminescence light reduction in vaccinated mice at 48 h post‐viral challenge compared to that in non‐vaccinated mice, with a reduction extent of “Lap@C‐C” > “C‐C” > “Lap.” In addition, the Lap@C‐C‐adjuvanted group was the only group that showed significant light reduction as early as 24 h post‐viral challenge.

To further elucidate the initiated T‐cell immune responses, mice were euthanized 3 days after the viral challenge, and the liver lymph nodes (LLNs) responsible for liver immune surveillance were separated for analysis. Both the LLN size (Figure 6e) and the number of contained lymphocytes (Figure 6f) of “Lap@C‐C”‐adjuvanted mice were remarkably larger and higher, respectively than those of their counterparts. In addition, the “Lap@C‐C”‐adjuvanted group had the highest proportion of OVA_257–264_‐specific CD8^+^ T‐cells in LLNs utilizing the MHC‐peptide tetramer (Figure 6g). Moreover, CD8^+^ T‐cells in the “Lap@C‐C”‐adjuvanted group expressed significantly higher activation marker levels of CD25, CD44, CD69, and CD107α (Figure 6h) as well as higher levels of intracellular cytokines of TNF‐α and interferon‐γ (Figure 6i) than those in the “C‐C”‐ or “Lap”‐adjuvanted groups.

Concurrently, the liver sections of “Lap@C‐C”‐adjuvanted mice showed the largest amount of inflammatory cell infiltration (Figure 6j, upper panel) and CD8^+^ T‐cell recruitment (Figure 6j, k lower panel) to eradicate Ad‐OVAp‐Fluc‐infected hepatocytes, which was consistent with the results of viral clearance shown in Figure 6c. Overall, these results confirmed that antigen‐pulsed DC vaccines provide effective protectivity from antigen re‐challenge. Most importantly, the results also showed that “Lap@C‐C,” as the optimized adjuvant, is superior to the conventional adjuvant “C‐C” or the monocomponent Lap in aiding DCs with initiating antigen‐specific CD8^+^ T‐cells. Notably, the PEG modification of Lap remarkably mitigated the adjuvant effect of “Lap@C‐C” (Figure 6c–k), indicating that Lap‐induced calcium flickering via lysosomal reprogramming is indeed a major driver of the enhanced immune efficacy of adoptive DC vaccines.

Hepatitis B virus (HBV) infection poses a significant threat to public health, with an estimated 296 million chronically infected patients and more than 800000 deaths worldwide.^[^ ^45^ ^]^ The first‐line therapies, pegylated interferon and nucleos(t)ide analogs, can inhibit HBV replication but do not eradicate the virus and rarely clear hepatitis B surface antigens (HBsAgs).^[^ ^46^ ^]^ Due to several inhibitory mechanisms, both DCs and HBV‐specific T‐cells are severely dysfunctional during chronic HBV infection.^[^ ^47^ ^]^ Therefore, functionally competent adoptive DC vaccines are expected to rescue the impaired antiviral immunity and disrupt HBV‐induced immune tolerance.

**Figure 7:**
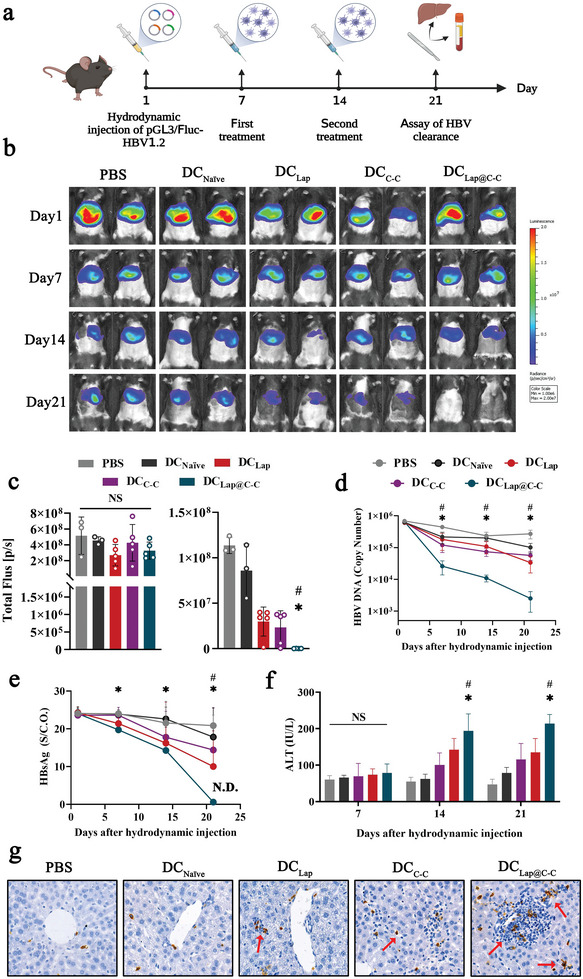
Evaluation of Lap@C‐C adjuvanted DC vaccine against hepatitis B virus (HBV). a) Construction schedule of visualized HBV clearance mouse model. b, c) Representative images and statistical analysis of fluorescence intensity in the mouse liver after hydrodynamic injection of pGL3/Fluc‐HBV1.2. d, e) Assay of serum HBV DNA copy number and hepatitis B surface antigen (HBsAg) titers. f) Serum alanine aminotransferase activity in mice. g) Infiltration of CD8^+^ T cells into the liver detected by immunohistochemical staining. Data represent mean ± SD (n = 5). *P < 0.05 DC_Lap@C‐C_ group compared with the DC_Naïve_ group. #*P* < 0.05 DC_Lap@C‐C_ group compared with the DC_C‐C_ group. Representative results from two or three replicates are shown.

The finding that Lap@C‐C‐adjuvated DCs could induce robust antigen‐specific antiviral CD8^+^ T‐cell immune responses encouraged the investigation of their therapeutic effects in clearing chronic HBV infections in this study. A mouse model of long‐term HBV expression was constructed by hydrodynamically transferring HBV 1.2 DNA (HBV genome) and Fluc^+^ (a marker gene) into mouse hepatocytes to simulate chronic HBV infection (**Figure** **7a**). HBV DNA and HBsAg persisted in the serum for >6 months (data not shown), and HBV clearance in hepatocytes can be detected directly using BLI (Figure 7b). The established HBV mouse model was injected twice with hepatitis B core antigen‐pulsed DCs stimulated with various adjuvants for virological and serological analyses. Randomized grouping ensured that the HBV infection was maintained at a comparable level in all groups before DC therapy (Figure 7c, left panel). Seven days after the second DC injection, HBV clearance was imaged, and showed an ≈ 6‐fold light reduction in “C‐C”‐adjuvanted mice compared to those injected with naive DCs (Figure 7c, right panel). In sharp contrast, the light signal in the “Lap@C‐C”‐adjuvanted group diminished by ≈870‐fold. In addition, this group showed a 260‐fold reduction in DNA load, with a clearance of >99.6% of the viral copies in the serum (Figure 7d). Moreover, the serum HBsAg levels decreased rapidly in Lap@C‐C‐adjuvated mice, reaching an undetectable level 7 days after the second DC therapy; whereas those in “C‐C”‐ or “Lap”‐adjuvanted mice remained high (Figure 7e). Consistently, the alanine aminotransferase level was simultaneously elevated in DC‐vaccinated mice owing to the killing of HBV‐infected hepatocytes,^[^ ^48^ ^]^ sharing a similar tendency with serum DNA or HBsAg detection (Figure 7f). Moreover, considerably higher CD8^+^ T‐cell infiltration was observed in the inflammatory lesions of the liver sections of mice immunized with Lap@C‐C‐adjuvated DCs compared to that of their counterparts, confirming that a robust antigen‐specific cytolytic response was initiated (Figure 7g). Collectively, these results proved the feasibility of employing DC vaccines as a therapeutic strategy for the control and clearance of HBV infection. In particular, Lap@C‐C, as the optimized adjuvant, possesses a huge advantage over conventional adjuvants (C‐C) in activating the anti‐HBV T‐cell immune response. Importantly, all mice that received Lap@C‐C‐adjuvanted DCs finally underwent HBsAg seroconversion, which is rarely achieved in first‐line therapies; thus, Lap@C‐C has great potential as a novel DC vaccine adjuvant for anti‐HBV therapy.^[^ ^45^ ^]^

Similar to all nanomaterials designed for biomedical applications, Lap must undergo sufficient preclinical toxicity tests to accelerate translation. Nevertheless, as an adjuvant used for in vitro‐prepared DC vaccines, the vast majority of Lap is removed before DC administration in vivo, and the less internalized Lap is mainly retained in the lysosomes of DCs and is further degraded into non‐toxic magnesium silicate, sodium, and potassium salts (Table S1, Supporting Information). Furthermore, subcutaneous inoculation of DC vaccines can prevent their direct entry into the circulatory system to a large extent, thereby greatly reducing potential hazards. Within 1 week of the last vaccination, the whole blood counts showed no statistically significant differences from those of untreated mice (Table S2, Supporting Information). No obvious histological lesions were observed in the heart, liver, spleen, lungs, kidneys, brain, or intestines (Figure S20, Supporting Information). Although further studies are warranted, Lap can be considered a potentially efficacious and safe nanoadjuvant.

## 3 Conclusion

**Figure 8:**
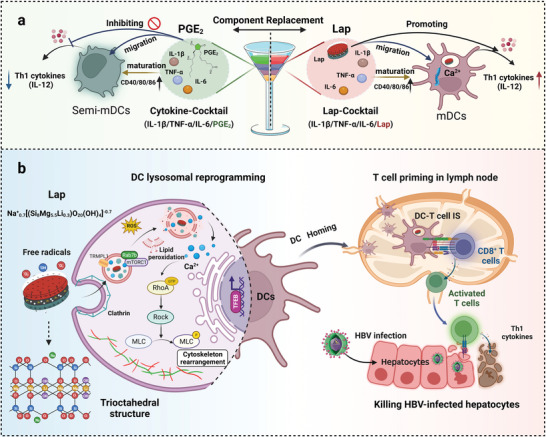
Schematic of Lap promoting DCs migration and HBV treatment. a) Lap@C‐C could overcome the limitations of prostaglandin E_2_ (PGE_2_) and stimulate the maturation of DCs and the secretion of T helper 1 (Th1) cytokines, which in turn primed CD8^+^ T cells. b) Lap promoted the release of Ca^2+^ from lysosomes, thereby promoting DCs homing to lymph nodes to activate cytotoxic T cells and clear HBV‐infected hepatocytes.

The overall mechanisms of the Lap action and the Lap@C‐C construction process are shown in **Figure** **8**. The morphology of Lap and its free radicals determines the interaction patterns between Lap and DCs. DCs exhibited high affinity for Lap nanosheets (≈28 nm in diameter and ≈1 nm thick), resulting in Lap internalization into lysosomes via clathrin‐mediated phagocytosis. Lap‐generated free radicals could induce DC lysosomal reprogramming, which involved increased LMP followed by calcium flickers, facilitating cytoskeletal reorganization, and further increasing DC migration. Simultaneously, LMP led to antigen escape from lysosomes and accelerated antigen presentation in DCs. These results lay a foundation for Lap‐enhanced DC‐T‐cell interactions and antigen‐specific CD8^+^ T‐cell activation. A novel C‐C optimization scheme was proposed by replacing PGE_2_ with Lap to constitute Lap@C‐C, and DCs stimulated by Lap@C‐C possessed high migratory potential and produced higher levels of IL‐12p70 than the current gold standard C‐C. In addition, the Lap@C‐C‐adjuvanted DC vaccine showed a superior ability to induce an antiviral CD8^+^ T‐cell response and greatly enhanced the clearance of HBV in vivo, resulting in > 99.6% viral DNA clearance and successful HBsAg seroconversion. To the best of our knowledge, this is the first study to systematically investigate the immunostimulatory potential of Lap and uncover a novel regulatory axis of lysosome reprogramming: calcium flicker–cytoskeleton organization. These findings not only provide beneficial support for personalized DC therapy but also shed light on the development of universally used DC‐targeted innovative nanoadjuvants.

## 4 Experimental Section

All experiments were conducted by the National Institutes of Health Guide for the Care and Use of Laboratory Animals and were approved by the Committee on Animal Care and Use of the Academy of Military Medical Sciences (Approval No.: IACUC‐DWZX‐2022‐600).

All experimental data were statistically analyzed using SPSS (version 26.0), expressed as mean ± standard deviation (mean ± SD), and plotted using GraphPad Prism 9.2. One‐way analysis of variance or Student's t‐test was used to determine significant differences. *P* < 0.05 was considered to indicate statistical significance.

Details of the used reagents, antibodies, and methods can be found in the Supporting Information.

## Conflict of Interest

The authors declare no conflict of interest.

## Supporting information

Supporting Information

Supplementary Video 1

Supplementary Video 2

Supplementary Video 3

Supplementary Video 4

Supplementary Video 5

## Data Availability

The data that support the findings of this study are available from the corresponding author upon reasonable request.
