# Supplementary material for: Laponite Lights Calcium Flickers by Reprogramming Lysosomes to Steer DC Migration for An Effective Antiviral CD8+ T‐Cell Response
Source: Adv Sci (Weinh). 2023 Aug 28;10(30):2303006. doi: 10.1002/advs.202303006 (PMC10602536; doi:10.1002/advs.202303006)
Supplement: Supplementary file 1 — Supporting Information [file ADVS-10-2303006-s004.pdf]

ADVANCED SCIENCE
Open Access

Supporting Information

for *Adv. Sci.*, DOI 10.1002/advs.202303006

Laponite Lights Calcium Flickers by Reprogramming Lysosomes to Steer DC Migration for An Effective Antiviral $CD8^+$ T-Cell Response

Chenyan Li, Yangyang Hou, Minwei He, Liping Lv, Yulong Zhang, Sujing Sun, Yan Zhao, Xingzhao Liu, Ping Ma, Xiaohui Wang, Qianqian Zhou* and Linsheng Zhan**

Supporting Information

Laponite Lights Calcium Flickers by Reprogramming Lysosomes to Steer DC Migration for an Effective Antiviral CD8$^+$ T-Cell Response

Chenyan Li[1,2,#], *Yangyang Hou*[1], *Minwei He*[1], *Liping Lv*[1], *Yulong Zhang*[1], *Sujing Sun* [1], *Yan Zhao*[1], *Xingzhao Liu, Ping Ma*[1], *Xiaohui Wang*[1]*, *Qianqian Zhou*[1]*, *Linsheng Zhan*[1,2]*

1: Institute of Health Service and Transfusion Medicine, Beijing 100850, P. R. China;
2: BGI college, Zhengzhou University; Henan Institute of Medical and Pharmaceutical Science, Zhengzhou University, Zhengzhou 450001, P. R. China;
*To whom correspondence should be addressed.
Tel.: +86-10-66931292
Fax: +86-10-66931292
E-mail: amms91@126.com; zhouqianvv521@126.com; lovechina1980@163.com.

Methods

Characterization of Lap

The Lap powder was purchased from BYK (USA) and was dispersed in ultrapure water and sonicated (15 min at 100 W) for the subsequent experiments. Lap morphology was characterized using atomic force microscopy (AFM, Bruker Dimension ICON, Germany), transmission electron microscopy (TEM, Hitachi H-7650, Japan), and scanning electron microscopy (SEM, Hitachi SU8220, Japan). The hydrodynamic size and surface charge of the AuNPs in water or culture medium were measured using the Zetasizer Nano ZS90 system (Malvern Instruments, England). Detection of free radicals in Lap was undertaken using electron paramagnetic resonance spectroscopy (EPR, Bruker ELEXSYS E500, Germany). Accurate quantification of Si in cells was performed by inductively coupled plasma mass spectrometer (ICP-MS) analysis (PerkinElmer NexION 300X, USA) through wet digestion.

Mice

Male 6–8-week-old wild-type C57BL/6J mice were purchased from the Beijing Vital River Laboratory Animal Technology Co.. L2G85.C57BL/6 transgenic mice were obtained by backcrossing L2G85 (FVB) mice expressing Firefly luciferase (Fluc) into C57BL/6J mice and used at stage N7. These mice, expressing $Fluc^+$, were backcrossed with C57BL/6J mice, and were used at Phase N7. tdTomato and OT-I transgenic mice were purchased from the Model Animal Research Center of Nanjing University. All animals were housed under SPF conditions at the National Beijing Center for Drug Safety Evaluation and Research (NBCDSER) and kept in the facility for at least 1 week before participating in experiments.

Generation of Bone marrow-derived immature DCs

Bone marrow monocytes were isolated from the femurs of C57BL/6J mice or L2G85.C57BL/6 transgenic mice and cultured in RPMI-1640 medium containing 10% fetal bovine serum, 10 ng/mL murine GM-CSF, and 5 ng/mL murine IL-4. The previous medium was replaced by 2 mL fresh growth medium with GM-CSF and IL-4 on Day 3 and 5. Bone marrow-derived DCs were obtained on day 6 and used as immature DCs. The mouse bone marrow-derived immature DC line JAWS Ⅱ was purchased from the American Type Culture Collection (ATCC) and cultured in RPMI-1640 medium containing 20% fetal bovine serum and 5 ng/mL murine GM-CSF. The cultures were kept at 37 ºC in a humidified incubator under a 5% CO_2 atmosphere.

FACS analysis

DCs (1×10^6 cells/mL) were incubated with different doses of Lap (25 μg/mL, 50 μg/mL, and 100 μg/mL) for 12 h in RPMI-1640 complete medium in 6-well culture plates. Flow cytometry was used to detect the expression of costimulatory molecules (CD40, CD80, CD86, and MHC II) and chemokine receptors (CCR5, CCR7, and CXCR4) in the DCs. 1×10^6 cells were harvested by centrifugation at 400 rcf for 5 min, and then stained with the corresponding antibodies for 20 min at 4 °C. The reaction was stopped by washing twice with PBS and the cells were subjected to FACS analysis in an 8-color FACS Calibur (BD Biosciences, Mountain View, CA). For apoptosis detection, cells were stained with Annex Ⅴ (BD PharMingen). To detect antigen-presenting ability, DCs were stained with anti-H-2Kb-SIINFEKL after co-incubation with $OVA_{257\text{-}264}$ (1 μg/mL) for 12 h. Thereafter, DCs were collected and washed twice to remove the free antigens before use. Total cell populations were gated by CD11c for all the analyses. Data were collected and analyzed using FlowJo V10 software. The isotype contrast for each antibody was used as the gate control.

Cytokine Secretion Assay

For cytokine secretion assays, the medium supernatant were harvested and centrifuged at 500 rcf for 5 min at 4 °C. Supernatants were used to measure pro-inflammatory cytokines IL-6, IL-12p70, IL-1β, and TNF-α secretion with commercially available ELISA kits (Dakewe, Shenzhen, China) following the producer’s protocol. The optical density of each well was determined using a microplate reader (Molecular Devices, USA) at 450 nm.

Biocompatibility evaluation of Lap

For the MTT assay, DCs (2×10^5 cells/mL) were treated with different doses of Lap (25 μg/mL, 50 μg/mL, 100 μg/mL, and 200 μg/mL) for 12 h in RPMI-1640 complete medium in 96-well culture plates. and subsequently assayed using the Vybrant® MTT-cell Proliferation Assay Kit (Thermo Scientific, USA) following the producer’s protocol. For apoptosis detection, DCs were treated with different doses of Lap (25 μg/mL, 50 μg/mL, and 100 μg/mL) and subsequently assayed using Annexin V Apoptosis Detection Kit (BD Pharmingen) following the producer’s protocol. JAWSII

In vitro migration analysis of DCs

DCs derived from tdTomato transgenic mice were cocultured with different doses of Lap (25 μg/mL, 50 μg/mL, and 100 μg/mL). 2×10^5 DCs were seeded in 8-well Nunc™ Lab-Tek™ Chambered Coverglass (Thermo Scientific, USA), and the velocity and migration distance per unit time were monitored using a live cell time-lapse imaging system (PerkinElmer, USA). Statistical analysis was performed using Volocity software.

Immunofluorescence staining and confocal microscopy

Cytoskeletal rearrangement of DCs was measured using an immunofluorescence assay after different treatments. 1×10^6 DCs subjected to different treatments were adhered to a 35mm confocal dishe at 37 °C for 2 h, fixed with 4% paraformaldehyde (Merck, Schwalbach, Germany) for 30 min, and permeabilized with 0.15% Triton X-100 in PBS (PBST) for 15 min at room temperature. The cells were labeled with 10 μg/mL rabbit anti-β-tubulin antibody (Abcam, ab179513) in 2% BSA in PBS at 4°C overnight, and then washed twice with PBS for 5 min each. After washing, the cells were incubated with 2% BSA in PBS for 30 min at room temperature. After blocking, DCs were incubated with AF594-conjugated phalloidin (Invitrogen, A12381), DAPI and 10 μg/mL AF488-conjugated goat anti-rabbit IgG (Abcam, ab150077) to visualize F-actin and β-tubulin. After incubation, cells were washed three times in PBS for 10 min each, and then dishes were mounted with the Prolong Gold Antifade reagent (Invitrogen) and observed under a laser confocal microscope (PerkinElmer, USA).

Lysosomal membrane permeabilization assay

Acridine orange (AO) (sigma, USA) was performed to assess the Lysosomal membrane permeabilization (LMP). 1×10^6 DCs subjected to different treatments were adhered to a 35mm confocal dishe at 37 °C for 2 h. DCs were incubated by AO (5 μg/mL) for 20 min at 37°C. After washing, DCs were visualized using a Zeiss LSM-900. The excitation wavelength was 488 nm, while the data were obtained at two separate emission wavelengths (505-560 nm, 590-690 nm). The decreased red fluorescence and enhanced green fluorescence were the sign of LMP.

For galectin puncta assay, 1×10^6 DCs subjected to different treatments were replated on a well in a 35mm confocal dishe at 37 °C for 2 h. After replating, DCs were fixed with 4% paraformaldehyde (Merck, Schwalbach, Germany) for 30 min, permeabilized with 0.15% Triton X-100 in PBS (PBST) for 15 min at room temperature, and then washed twice with PBS for 5 min each. After washing, the cells were incubated with 2% BSA in PBS for 30 min at room temperature. After blocking, cells were incubated with the mixture of 10 μg/mL rabbit anti-LAMP1 pAb and 10 μg/mL mouse anti- galectin-3/LGALS3 mAb (Abclonal, A13506) in 2% BSA in PBS at 4°C overnight. The cells were washed three times in PBS for 10 min each, and then incubated with the mixture of DAPI, 10 μg/mL Alexa Fluor 647 anti-mouse IgG and 10 μg/mL Alexa Fluor 488 anti-rabbit IgG (Abclonal, AS053) in 2% BSA in PBS for 2 h at room temperature. After incubation, cells were washed three times in PBS for 10 min each, and then visualized using a Zeiss LSM-900.

Western blot test

The cells were washed in ice-cold PBS, lysed by incubation for 30 min on ice in RIPA buffer supplemented with protease inhibitor, phosphatase inhibitor, and then centrifuged at 18,000 rcf for 20 min at 4°C. The supernatants were heated for 10 min at 97°C in LDS sample buffer, resolved by SDS-PAGE, and electrophoretically transferred to PVDF membranes (Invitrolon PVDF; Thermo Fisher Scientific, LC2005). After blocking with Dulbecco's phosphate-buffered saline (DPBS; Life Technologies, 14190-094) containing 5% milk and 0.1% Tween-20 (Sigma-Aldrich, 274348), the membranes were incubated overnight with rabbit anti-TFEB (Abcam, ab264424, 1:1000), rabbit anti-Rab7b (Abcam, ab193360, 1:1000) antibodies and reacted with HRP-coupled rabbit IgG (Abcam, ab6721, 1:500) for 1 h at room temperature. GAPDH was used as a loading control. Bound antibodies were detected with Clarity Western ECL Substrate (Bio-Rad, 170-5061) using a Odyssey XF imaging system (LI-COR, USA).

Identification of the Lap and DC interaction pattern

To explore the internalization of Lap, DCs derived from tdTomato transgenic mice were co-incubated with FITC-labeled Lap (50 μg/mL) for 12 h, with or without methyl-β-cyclodextrin (M-β-CD; caveolin inhibitor, MCE, HY-101461, 5 mM), chlorpromazine hydrochloride (CPZ; clathrin inhibitor, MCE, HY-B0407A, 1 μM), and ethyl-isopropyl amiloride (EIPA; macropinocytosis inhibitor, MCE, HY-101840, 15 μM), followed by colocalization analysis using CLSM. Time-lapse imaging of Lap internalization was performed using a high-speed confocal microscope (Oxford Instruments, UK). DCs stained with CellTracker™ Deep Red (Thermo Scientific, C34565, 5μM) were co-incubated with FITC-labeled Lap for 5 min, imaged for more than 2 h, and analyzed using Imaris 9.0.1 software.

Cytosolic Ca^{2+} measurements

For cytosolic Ca^{2+} imaging, DCs were replated on a well in a 35mm confocal dishe and washed 3 times with HBSS (Ca^{2+} free) . 2×10^5 DCs were loaded with 1 μM Fluo-4 AM (Dojindo Laboratories, F311) in HBSS for 30 min at 37°C. After incubation, cells were washed 3 times with HBSS to adequately remove residual Fluo 4-AM working solution, and then add 400μL HBSS to cover the cells. After DCs were treated by Lap (50 μg/mL) for 9 mins, imaging was performed at 20 ms per frame using a Zeiss LSM-900.

For image analysis, F_0 was defined as the background-subtracted fluorescence intensity at the initial time point, F denoted the background-subtracted fluorescence intensity at each time point, and $\Delta F / F_0 = (F - F_0) / F_0$ reflected the changes in cytosolic Ca^{2+} concentration of DCs during Lap internalization.

DC-T-cell interaction and in vitro T-cell priming

DCs derived from tdTomato transgenic mice were incubated with $OVA_{257-264}$ for 12 h and then incubated for another 12 h with different doses of Lap. $CD8^+$ T-cells were isolated and purified with mouse $CD8^+$ T Cell Isolation Kit (Miltenyi Biotec, 130-104-075) from OT-I transgenic mice following the producer's protocol. Then $CD8^+$ T-cells labeled with CellTracker™ Deep Red (Thermo Scientific, C34565, 5μM). Thereafter, the DCs were cocultured with $CD8^+$ T-cells at a 1:5 ratio (DCs: T-cells). Interactions between DCs and T-cells were monitored using a live cell imaging system (PerkinElmer, Massachusetts, USA), and colocalized areas visualized in dynamic images were analyzed using Imaris 9.0.1 software to calculate the DC-T-cell contact volume and duration.

To explore the role of Lap in DC-T-cell interactions, FITC-labeled Lap was added to the coculture system at a dose of 25 μg/mL. Thereafter, the attachment of Lap to DCs and T cells was observed using a live cell imaging system and measured using Imaris 9.0.1 software.

To detect in vitro activation of T-cells, Lap-pretreated and $OVA_{257-264}$-pulsed DCs were cocultured with $CD8^+$ T-cells isolated from OT-I transgenic mice at a ratio of 1:5 (DCs:T-cells) for 72 h. T-cell activation was detected by costaining for the markers CD8α with respective CD25, CD69, and CD107α. All samples were tested by FACS and analyzed by FlowJo V10, and the $CD8\alpha^+$ cell population was gated as T-cells.

Label-free quantitative proteomics analysis

Lap-treated and control DCs were collected with urea (300 μL of 8 M) and protease inhibitor (10% of lysate) added. After centrifugation at 14,000 rcf for 20 min, the supernatant was collected and the protein concentration was determined by Bradford's method, and a 100 μg aliquot of sample was taken for LC-MS/MS measurement. Label-free mass spectra were analyzed using MaxQuant software, and protein data were screened by the Beijing QLBio Company using the UniProt database.

Bioluminescence imaging of DC homing in vivo

$Fluc^+$ DCs (2×10^6) were injected into the footpads of wild-type mice and monitored every 24 h using an IVIS (PerkinElmer, USA). The migration rate was calculated by the ratio of fluorescence signal in the popliteal lymph nodes and inguinal lymph nodes to the signal on the footpads. Representative images and SI were captured and measured using Living Image 4.5.2 software.

Adenovirus challenge mouse model and in vivo T-cell priming

$OVA_{257\text{-}264}$-pulsed DCs were incubated with Lap and successively injected into the footpads of mice on days 1 and 7. On day 14, mice were challenged with Ad-OVAp-Fluc. Viral clearance was monitored every 24 h using IVIS. Mice were euthanized on day 16 and LLNs were collected for lymphocyte fractionation and pathological analysis.

Antigen-specific $CD8^+$ T-cell activation was detected by staining lymphocytes with CD8α and T-Select H-2 Kb $OVA_{257\text{-}264}$ tetramers (MBL, Japan). Subsequently, cell activation and intracellular cytokine release were detected by staining CD8α with respective CD25, CD69, CD107α, TNF-α, and IFN-γ. After staining, the cells were washed twice prior to FACS analysis.

Anti-HBV evaluation based on HBV mouse model

On day 1, a plasmid carrying HBV overfull-length 1.2 DNA and $Fluc^+$ (pGL3/Fluc-HBV1.2) was hydrodynamically injected into mice to establish a model of HBV infection. DC vaccines were administered via footpads on days 7 and 14. HBV clearance was monitored at 1 week intervals using IVIS, and the mice were euthanized 7 days after the secondary vaccination for virological, serological, and toxicological analyses.

Analysis of Lap degradation products

The lysosomes of DCs were extracted using the Lysosome Enrichment Kit (Thermo Scientific, USA) and the lysates were titrated into the Lap dispersion. The resulting product was freeze-dried, analyzed, and tested by the Shanghai WEIPU Testing Technology Group Ltd.

Figures and Tables:

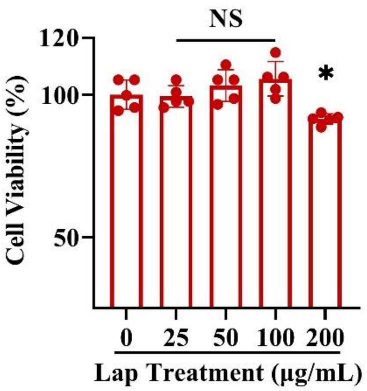

Figure S1. MTT analysis of DCs treated with Lap with dose ranging from 0-200 μg/mL. Statistics showed as mean ± SD (n = 5). NS: $P > 0.05$ compared with PBS group. *: $P < 0.05$ compared with 0 μg/mL.

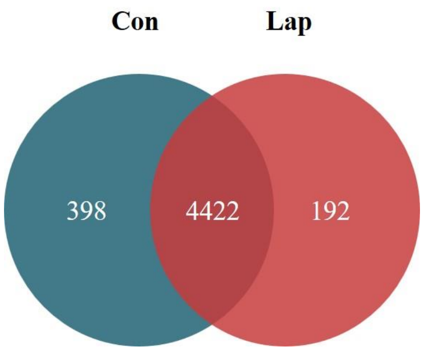

Figure S2. Venn diagram of differentially expressed proteins (DEPs) between Con-DCs (PBS-treated DCs) and Lap-DCs.

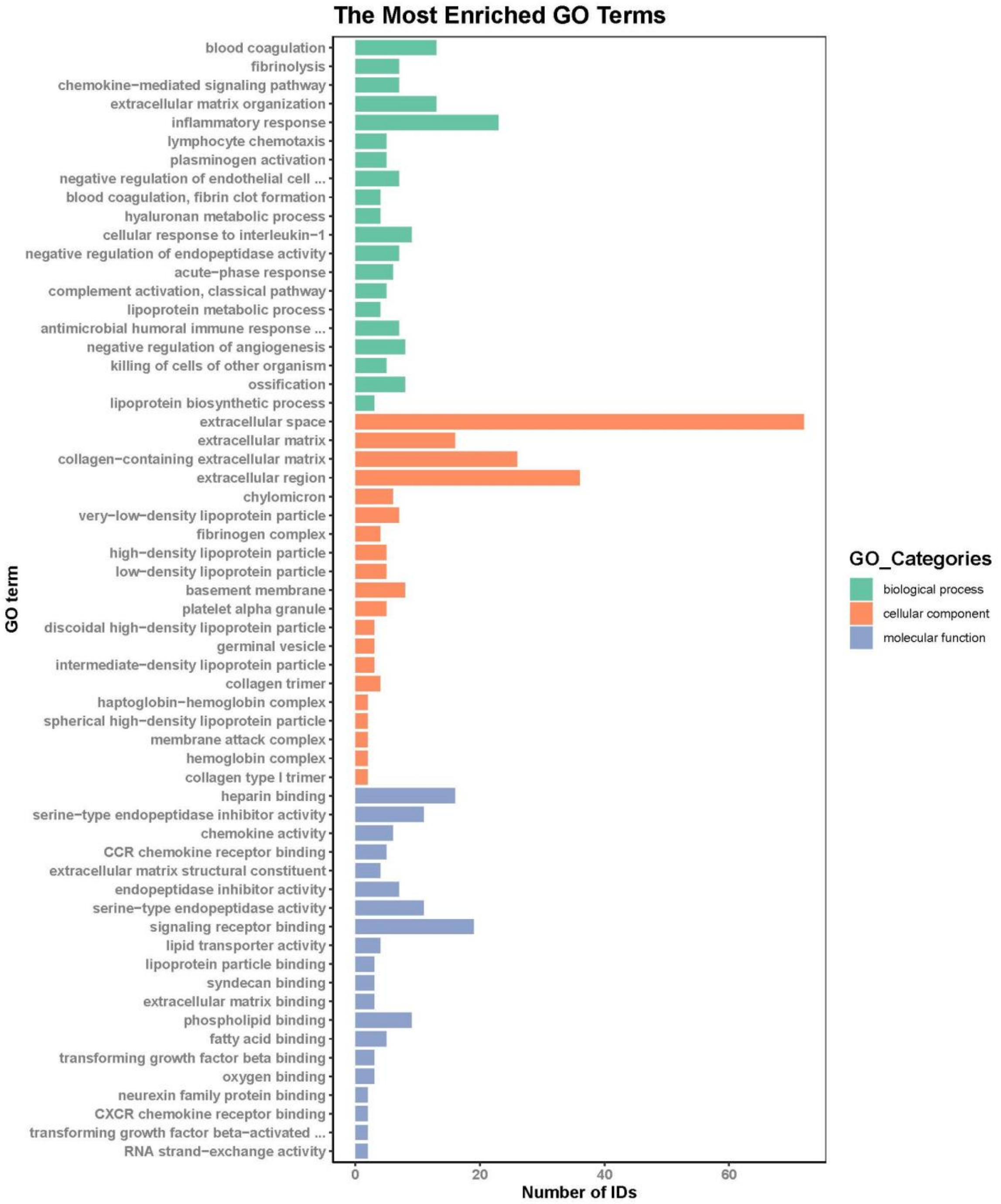

Figure S3. GO enrichment analysis for up-regulated DEPs. The bar color indicates the GO categories, and top 20 GO terms are shown.

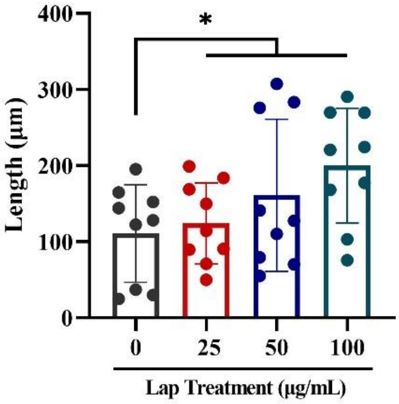

Figure S4. The migration distance of DCs treated with Lap with dose ranging from 0-100 μg/mL. Statistics showed as mean ± SD (n = 5). *: $P< 0.05$ compared with 0 μg/mL.

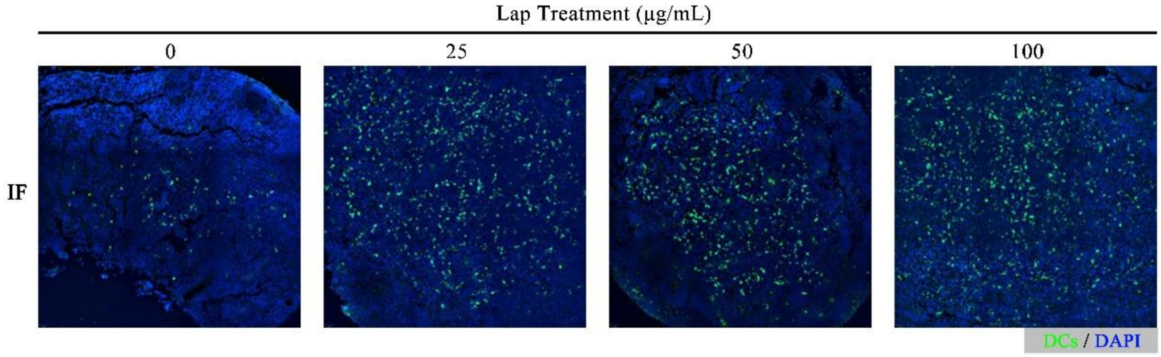

Figure S5. DC homing to popliteal lymph nodes (PLNs) detected by tissue staining. GFP expressing-DCs were treated with different doses of Lap and then were injected into the footpads of mice. After 72 h, mice were euthanized, and the PLNs were harvested for tissue section. Green: DCs; Blue: nuclei.

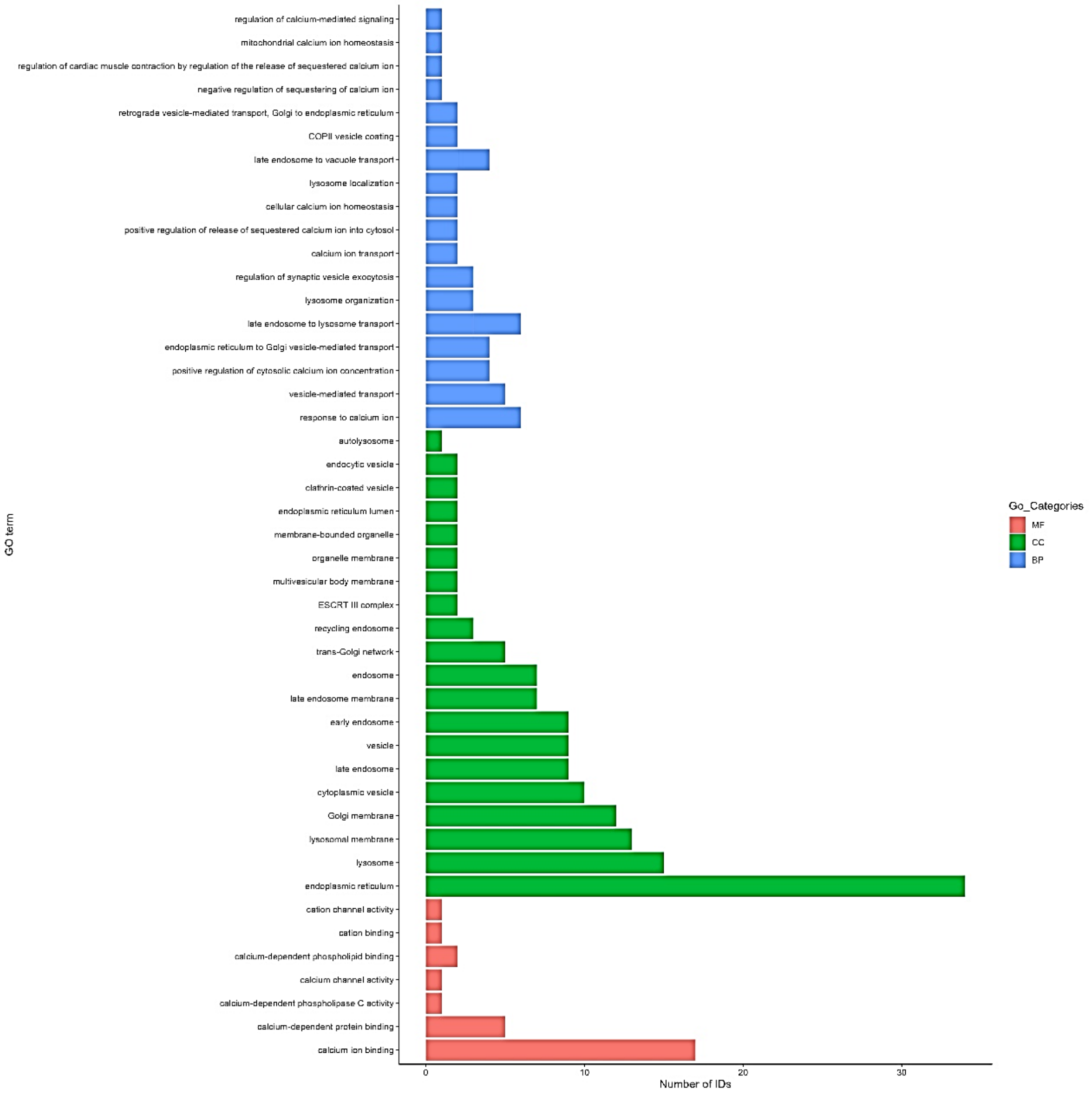

Figure S6. GO enrichment analysis for up-regulated DEPs involving vesicular transport and Ca^{2+} homeostasis. The bar color indicates the GO categories.

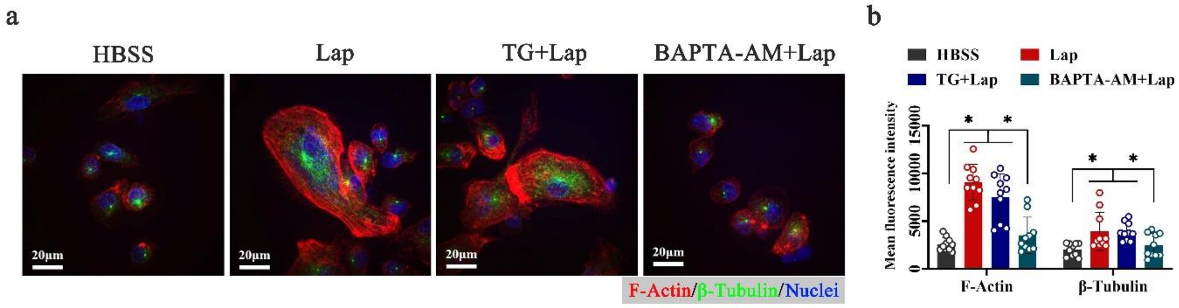

Figure S7. Cytoskeletal rearrangement of DCs after co-incubation with HBSS, Lap, TG+Lap and BAPTA-AM+Lap. a) Representative images of cytoskeletal rearrangement of DCs. Red: F-Actin; Green: β-Tubulin; Blue: Nuclei. The mean fluorescence intensity of F-actin and β-tubulin was measured and displayed as b). Data represent mean ± SD (n =10 statistics are shown

as mean ± SD (n = 21, "n" represents the number of cells observed per experimental group).). *$P < 0.05$ compared with the PBS group.

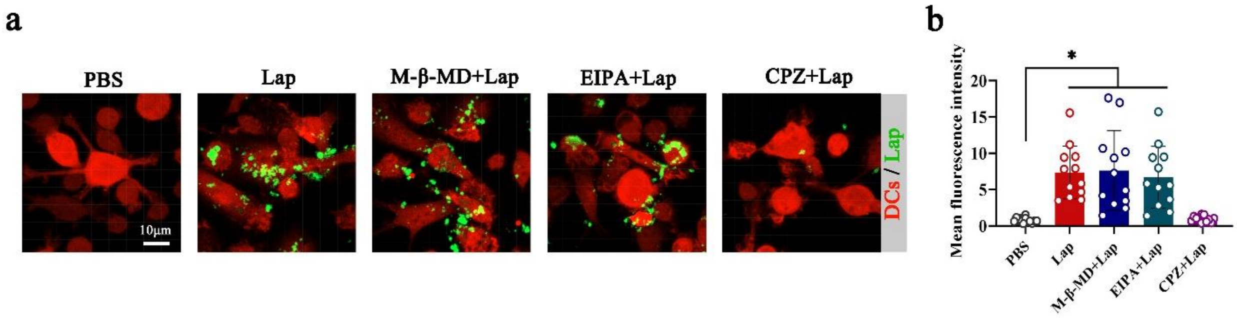

Figure S8. The internalization of Lap by DCs. **a)** CLSM images of DCs treated with Lap ± Methyl-β-cyclodextrin (M-β-CD, caveolin inhibitor, 5mM), Chlorpromazine hydrochloride (CPZ, clathrin inhibitor,1μM), and ethyl-isopropyl amiloride (EIPA, macropinocytosis inhibitor, 15μM). Red: DCs expressing tdTomato fluorescent protein; Green: FITC-labeled Lap. **b)** The mean fluorescence intensity of Lap internalization. Statistics are shown as mean ± SD (n = 12, "n" represents the number of cells observed per experimental group).

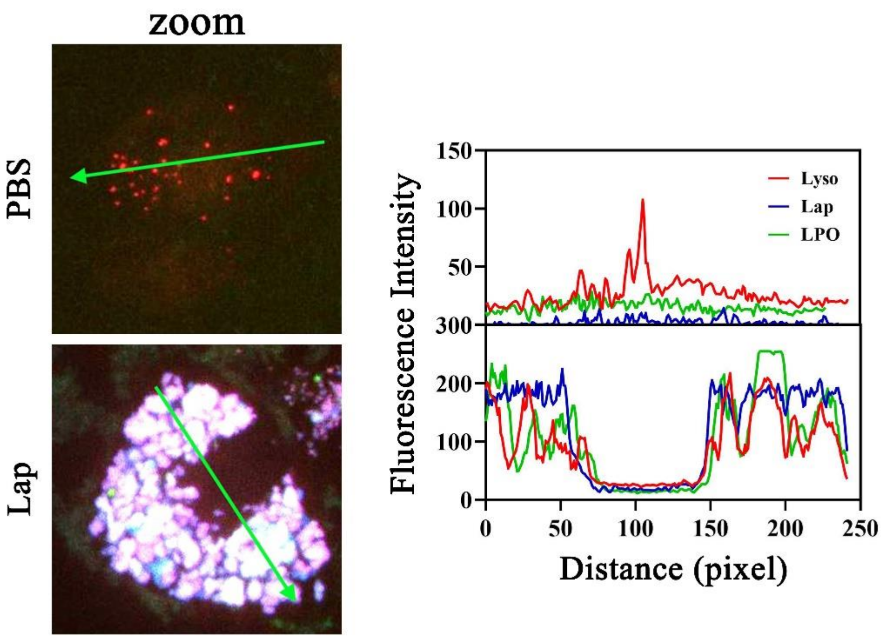

Figure S9. LPO accumulation in DCs after Lap co-incubation. Left panel: CLSM images of DCs treated with Lap for 1 h. Green: LPO indicator; Red: LysoTracker; Blue: rhodamine-labeled Lap. Right panel: fluorescence intensity statistics along the green arrows.

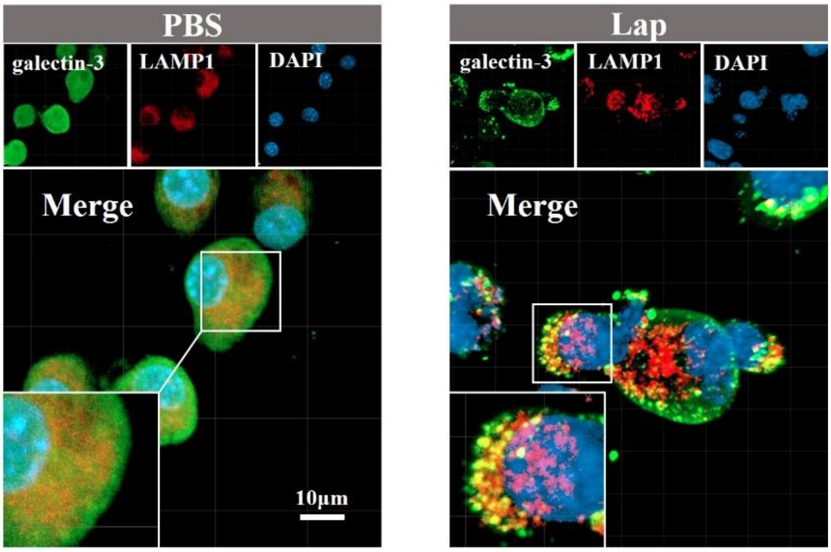

Figure S10. The detection of LMP with galectin-3 (LGALS3) puncta assay. Green: galectin-3 ; red: LAMP1; blue: nuclei; yellow: galectin-3 puncta formation positive for both galectin-3 and LAMP1.

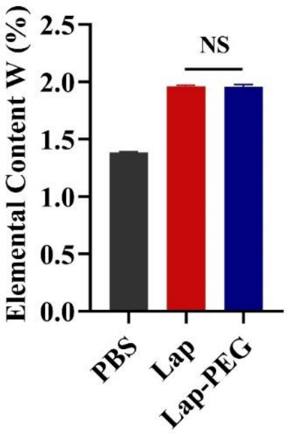

Figure S11. ICP-MS detection of elemental silicon in DCs. Statistics showed as mean ± SD (n = 3). NS: $P > 0.05$ compared with PBS group.

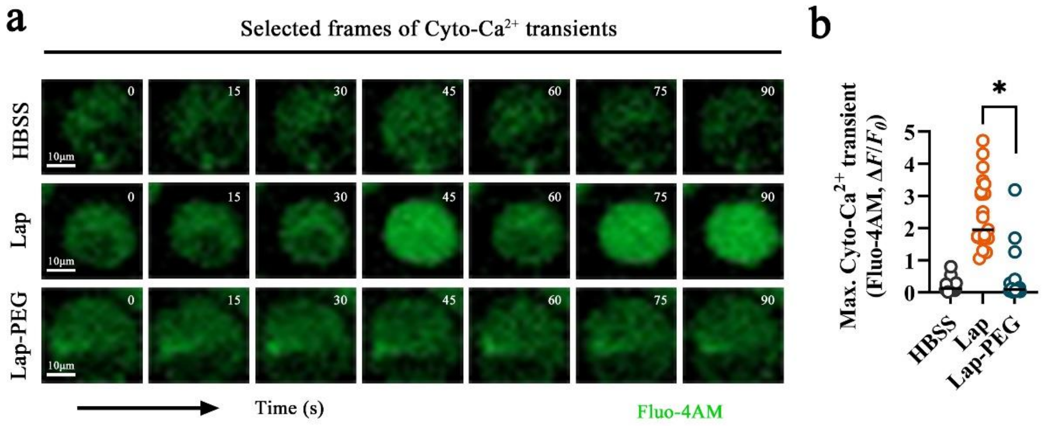

Figure S12. Dynamic monitoring of Cyto-Ca^{2+} in DCs. **a)** Representative Cyto-Ca^{2+} transients frames. **b)** Cyto-Ca^{2+} transients amplitudes were quantified by measuring the change in maximum fluorescence intensity of Fluo-4A. Statistics are shown as mean ± SD (n = 21, "n" represents the number of cells observed per experimental group).). *: $P < 0.05$ compared with Lap group.

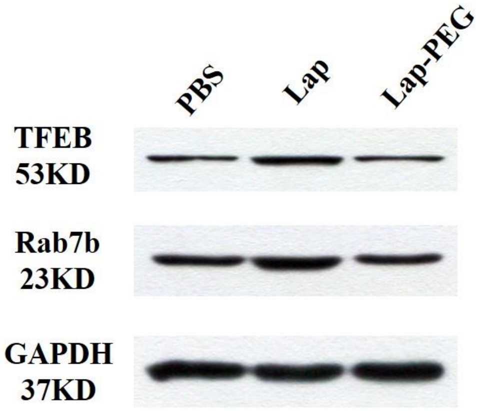

Figure S13. TFEB and Rab7b in DCs assessed with Western blot.

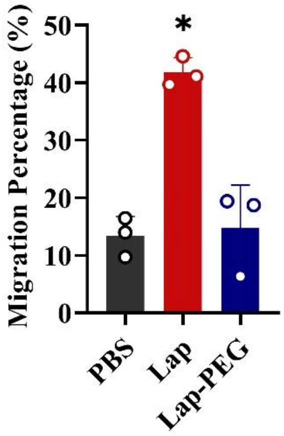

Figure S14. The statistical data of the homing percentage of tissue-resident DCs homing to lymph nodes. Data showed as mean ± SD (n = 3). *: $P < 0.05$ compared to PBS group.

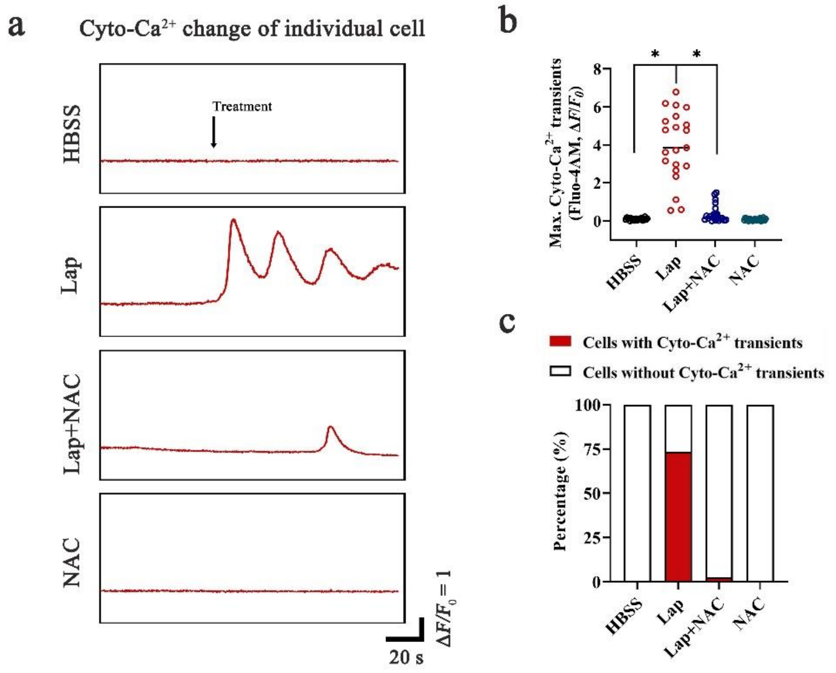

Figure S15. Dynamic monitoring of Cyto-Ca^{2+} in DCs. DCs were challenged with Lap ± N-acetylcysteine (NAC, ROS inhibitor, 5 mM). a) Representative tracking of the Cyto-Ca^{2+} dynamics of DCs labeled with Fluo-4AM. b) Cyto-Ca^{2+} transient amplitudes were quantified by measuring the change in maximum fluorescence intensity of Fluo-4AM. Statistics are shown as mean ± SD (n = 21, "n" represents the number of cells observed per experimental group). c) Percentage of DCs exhibiting Cyto-Ca^{2+} transients after different pretreatment. *$P < 0.05$ between the two groups indicated.

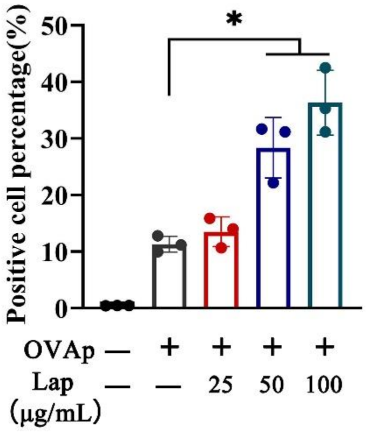

Figure S16. FCM assay of H-2Kb-SIINFEKL complexes on the surface of DCs. OVAp: ovalbumin 257–264. Data showed as mean ± SD (n = 3). *: $P < 0.05$ between the two groups indicated.

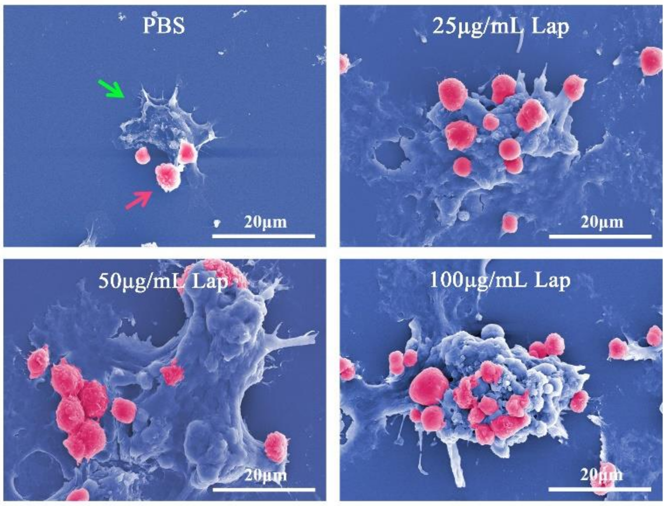

Figure S17. SEM images of DC-T cell contact. Green arrows: DCs; Red arrows: $CD8^+$ T cells derived from OT-I TCR transgenic mice.

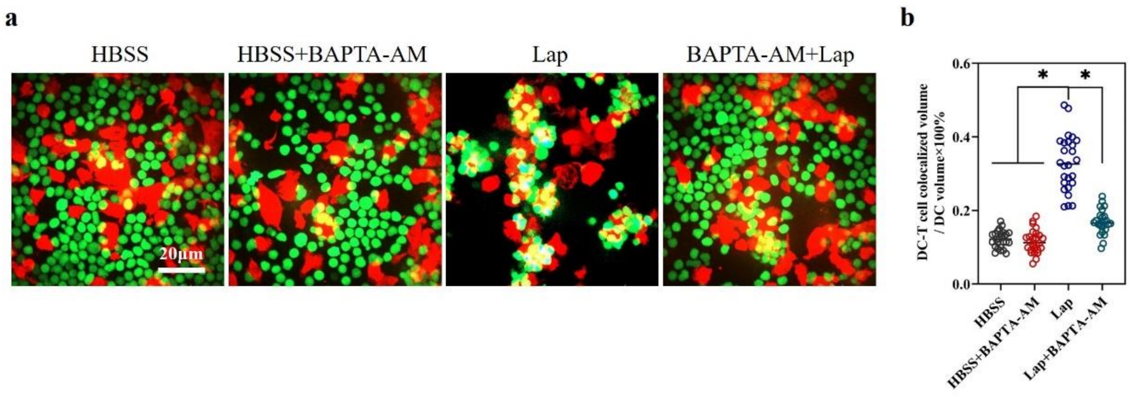

Figure S18. BAPTA-AM pretreatment impaired Lap enhanced DC-T cell IS formation. DCs from tdTomato transgenic mice were incubated with Lap (50 μg/mL). $CD8^+$ T cells from OT-I transgenic mice were purified by $CD8^+$ T Cell Isolation Kit (MiltenyiBiotec, Germany) and were labeled with the CSFE (Thermo Scientific, USA). Thereafter, DCs treated differently were cocultured with the labeled T cells at the ratio of 1:5 (DC: T cell). The interactions between DCs and T cells were monitored using a live cell imaging system (PerkinElmer, Massachusetts, USA). a) The colocalized areas of DC-T cells were analyzed using Volocity software. Red: DCs from tdTomato transgenic mice; Green: $CD8^+$ T cells from OT-I transgenic mice. b) The percentage of DC-T cell colocalization volume / DC volume. Data are mean±s.d.; n = 24 fields of view are shown; *$P < 0.05$ between the two groups indicated.

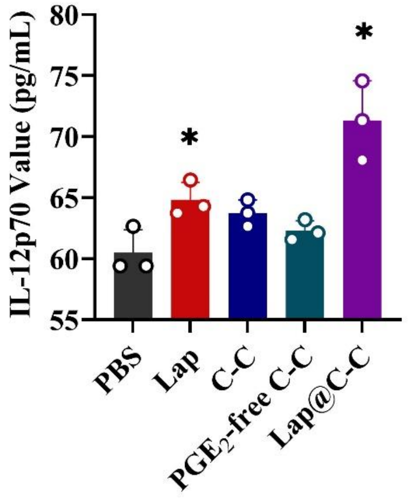

Figure S19. The level of IL-12p70 detected by ELISA assay. Data showed as mean ± SD (n = 3). *: $P < 0.05$ compared to PBS group.

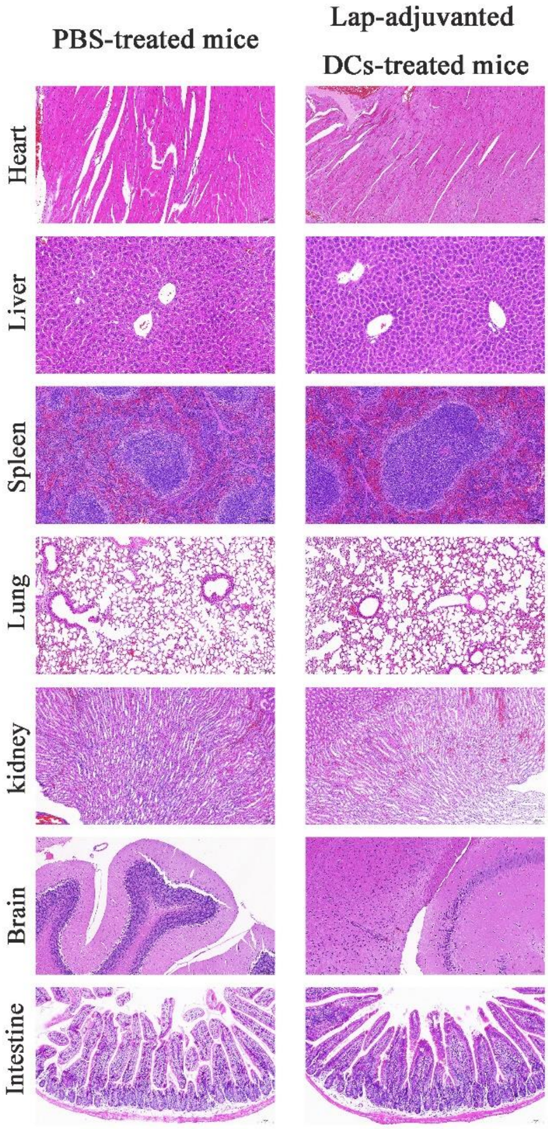

Figure S20. Hematoxylin & eosin (H&E) staining images of major organs of mice. A representative dataset from n=5.

Table S1. The degradation products of Lap

Chemical component	Mass fraction (wt%)
Magnesium silicate	~55.0-56.0
Silicic acid/silicates	~36.5-37.5
Sodium chloride	~5.0-6.0
Magnesium chloride	~1.0-2.0
Sulfate	~0.1-0.5

Table S2. The whole blood count of mice.

	PBS-treated mice	Lap-adjuvanted DCs-treated mice
WBC (10^9/L)	6.08 ± 0.606	5.68 ± 0.936
Lymph (10^9/L)	4.28 ± 0.572	3.66 ± 0.404
Mon (10^9/L)	0.14 ± 0.548	0.14 ± 0.548
Gran (10^9/L)	1.66 ± 1.033	1.88 ± 0.767
RBC (10^{12}/L)	8.126 ± 1.215	8.54 ± 0.625
HGB (g/L)	120.4 ± 20.72	125.4 ± 10.57
HCT (%)	35.82 ± 6.346	36.86 ± 3.153
MCV (fL)	43.96 ± 1.481	43.18 ± 0.698
MCH (pg)	14.72 ± 0.427	14.64 ± 0.305
MCHC (g/L)	335.8 ± 4.712	340 ± 2.449
RDW (%)	13.54 ± 1.941	12.06 ± 0.493
PLT (10^9/L)	624 ± 118.17	621.8 ± 154.427
MPV (fL)	4.32 ± 0.179	4.2 ± 0.1
PDW	16.24 ± 0.179	16.16 ± 0.288
PCT (%)	0.267 ± 0.041	0.260 ± 0.585

Data showed as mean ± SD (n = 5).

Supplementary Video 1. Dynamic monitoring of DCs internalizing Lap to lysosomes.; Red: DCs; Green: Lap; Blue: nuclei.

Supplementary Video 2. Dynamic monitoring of Cyto-Ca^{2+} in HBSS treated DCs; Green: Fluo-4AM.

Supplementary Video 3. Dynamic monitoring of Cyto-Ca^{2+} in Lap treated DCs; Green: Fluo-4AM.

Supplementary Video 4. Dynamic monitoring of Cyto-Ca^{2+} in TG and Lap treated DCs; Green: Fluo-4AM.

Supplementary Video 5. Dynamic monitoring of Cyto-Ca^{2+} in BAPTA-AM and Lap treated DCs; Green: Fluo-4AM.
